# Hypoxia-driven M2-polarized macrophages facilitate the epithelial-mesenchymal transition of glioblastoma via extracellular vesicles

**DOI:** 10.7150/thno.95766

**Published:** 2024-10-07

**Authors:** Liang Liu, Ran Wang, Aogesi Alifu, Yong Xiao, Yong Liu, Chunfa Qian, Mengjie Zhao, Xianglong Tang, Yandong Xie, Yan Shi, Yuanjie Zou, Hong Xiao, Kun Yang, Hongyi Liu

**Affiliations:** 1 Department of Neurosurgery, Affiliated Nanjing Brain Hospital, Nanjing Medical University, Nanjing 210029, China.; 2 Department of Neurosurgery, Second Affiliated Hospital of Soochow University, Suzhou 215004, China.; 3 Department of Neuro-Psychiatric Institute, Affiliated Nanjing Brain Hospital, Nanjing Medical University, Nanjing 210029, China.; 4 Department of Neurosurgery, Nanjing First Hospital, Nanjing Medical University, Nanjing 210006, China

**Keywords:** Glioblastoma, Hypoxia, TAMs, epithelial-mesenchymal transition, nanoplatform

## Abstract

**Rationale:** M2-like tumor-associated macrophages (TAMs) promote the malignant progression of glioblastomas. However, the mechanisms responsible for this phenomenon remain unclear.

**Methods:** RT-PCR, Western blot and flow cytometry were used to evaluate the polarization status of macrophages. RT-PCR, western blot or/and immunohistochemistry was used to determine the expression of circ_0003137, PTBP1, PLOD3 and epithelial-mesenchymal transition (EMT) markers. Transwell assay was used to assess migration and invasion ability of tumor cells. RNA sequencing, bioinformatic analysis and Pearson correlation coefficient was performed to explore the relation between PTBP1 and circ_003137/PLOD3. *In vivo* experiment was used to determine the role of sh-circ_0003137-loaded nanoplatform.

**Results:** Hypoxia promoted the polarization of macrophages towards M2-like TAMs in an HIF1α dependent manner. Then, M2-like TAMs could transport circ_0003137 enriched extracellular vesicles (EVs) to glioblastoma cells, upregulating circ_0003137 in glioblastoma cells. The circ_0003137 overexpression promoted the EMT of glioblastoma cells *in vitro* and *in vivo*. Mechanistically, circ_0003137 physically binds to polypyrimidine tract binding protein 1 (PTBP1), enhancing the stability of procollagen-lysine, 2-oxoglutarate 5-dioxygenase 3 (PLOD3) and promoting the EMT of glioblastoma cells. Moreover, a liposome-based nanoplatform that delivers shRNAs was established and used to encapsulate sh-circ_0003137. The fluorescence microscope tracer and cell co-culture assays demonstrated that the nanoplatform encapsulated with sh-circ_0003137 was stable and could penetrate the blood-brain barrier (BBB), finally reaching the central nervous system (CNS). The intracranial *in situ* tumor model showed that injecting the sh-circ_0003137-loaded nanoplatform via the tail vein significantly inhibited glioblastoma progression and improved the nude mice's survival.

**Conclusions:** Hypoxia can drive macrophage polarization towards M2-like TAMs. Polarized M2-like TAMs can transport circ_0003137 to glioblastoma cells through EVs. Then, circ_0003137 promotes the EMT of glioblastomas by targeting the PTBP1/PLOD3 axis. Hence, targeting circ_0003137 might be a novel therapeutic strategy against glioblastoma.

## Introduction

Glioblastoma is the most common central nervous system (CNS) primary tumor, accounting for about 26.5% 1, 2. The latest statistics show that glioblastoma incidence is 3-6 per 100,000, with hundreds of thousands of deaths yearly 3. Currently, the main treatment for glioblastoma is maximum tumor resection, followed by radiotherapy and chemotherapy. Despite the clinician's efforts, the prognosis for glioblastoma patients remains very poor, especially for glioblastoma multiforme (GBM) patients whose 5-year survival rate is only 5.5% and median survival time is only 14 months 4, 5. Therefore, exploring the mechanisms of glioblastoma genesis and development at the molecular level is imperative to formulate new treatment strategies.

Epithelia-mesenchymal transition (EMT) refers to the transformation of polar epithelial into stromal cells triggered by physiological or pathological conditions, which plays an important role in the progression of tumors, including glioblastoma 6, 7. During the EMT, due to loss of epithelial cell polarity, the adhesion ability to surrounding cells/stroma decreases while the migration and locomotion ability increase 8, 9. One of the main reasons for glioblastoma's poor clinical treatment is that glioblastoma cells undergo EMT during progression and acquire migration and invasion abilities, accelerating the malignant progression and difficult treatment 10. Therefore, the underlying mechanisms driving the EMT are crucial to search for novel targets related to glioblastoma diffusion and infiltration.

As one of the most abundant tumor microenvironment (TME) components, tumor-associated macrophages (TAMs) are closely related to tumor genesis 11. The M2-like subtype promotes tumor progression, including genetic instability, nourishing tumor stem cells, and acclimated protective adaptive immunity 12, 13. Also, studies have shown that M2-like TAMs promote the EMT of breast, lung, and gastric cancers 14, 15. He *et al.* reported that M2-like TAMs could facilitate the EMT of glioblastomas, shortening the survival of glioblastoma patients 16. However, the underlying mechanisms remained unclear.

Hypoxia is an important characteristic of the glioblastoma microenvironment and an important driving force for its malignant progression. Studies have shown that hypoxia promotes glioblastoma metastasis and deteriorates the prognosis of glioblastoma patients 17, 18. Until now, most studies have focused on hypoxia effects on glioblastoma parenchymal cells or glioblastoma stem cells (GSCs), and few reports are available on its effect on the polarization of TAMs, which infiltrate the glioblastoma microenvironment.

In the present study, we found that hypoxic TME can promote the polarization of macrophages towards M2-like TAMs. Polarized M2-like TAMs carry circ_0003137-enriched extracellular vesicles (EVs) to glioblastoma cells, leading to circ_0003137 upregulation. Then, circ_0003137 promotes the EMT progression of glioblastoma cells by targeting the PTBP1/PLOD3 axis, accelerating the malignant progression of glioblastoma. Additionally, animal experiments have shown that the sh-circ-0003137-loaded nanoplatform can effectively inhibit the EMT of glioblastoma cells and significantly improve the survival of tumor-bearing mice. Overall, we provided a reliable experimental basis for exploring circ_0003137 as a therapeutic target for glioblastoma in clinical practice.

## Materials & methods

### Bioinformatics analysis

The GSE131928 single-cell RNA-seq data and annotations were analyzed using the TISCH platform. The 'Seurat' V4 R package was used for subsetting, clustering, and non-linear dimensional reducing. Macrophage-related markers were visualized on a dimensional reduction plot using Seurat's 'FeaturePlot' function according to gene expression levels. The Cancer Genome Atlas (TCGA) glioblastoma expression dataset and metadata were collected from Xena and GlioVis platforms. The immune infiltration levels of TCGA glioblastoma cohorts were calculated using TIMER 2.0. Kaplan-Meier survival analysis was implemented using 'survival' and 'survminer' R packages.

The score of M2-like TAMs is calculated using the GSVA method. Specifically, a set of authoritative TAM cell markers was collected from Cell Marker 2.0, and GSVA algorithm was applied to score each sample in the expression matrix 19.

The PTBP1/PLOD3 expression levels were visualized using heatmaps generated from TCGA and CGGA datasets, selecting seven clinical features (histology, grade, IDH status, 1p/19q co-deletion, MGMT status, age and gender) for analysis. Samples were sorted and unsupervised clustered was performed based on the high to low expression levels of PTBP1/PLOD3.Violin plots were used to separately display clinical features with statistical significance. Furthermore, the TCGA and CGGA datasets were used to assess the impact of high and low PTBP1/PLOD3 expression on the survival prognosis of patients.

### Clinical specimens

Clinical specimens, including normal brain (n = 10) and glioblastoma (n = 30) tissues, were collected in the Department of Neurosurgery, Affiliated Nanjing Brain Hospital, Nanjing Medical University and Department of Neurosurgery, Second Affiliated Hospital of Soochow University. The pathological diagnosis of glioblastoma was made independently by two senior pathologists according to the World Health Organization (WHO) diagnostic criteria. Written informed consent was obtained from patients or their clients. The research ethics committee of the Affiliated Nanjing Brain Hospital, Nanjing Medical University, approved this study.

### Cell culture and transfection

U118, U251, T98G, A172, and LN229 glioblastoma cell lines were purchased from Procell (Wuhan, China) and cultured with Dulbecco's Modified Eagle Medium (DMEM, Gibco, USA) supplemented with 10% fetal bovine serum (FBS, scienCell, USA) in a 37 °C, 5% CO_2_ incubator. THP-1 was purchased from Procell and cultured in RPMI-1640 (Gibco, USA) supplied with heat-inactivated 10% FBS in a 37 °C, 5% CO_2_ incubator. The oxygen concentration in the incubator (Thermofisher, USA) was set to 20% to simulate normoxia and 1% to simulate hypoxia.

The THP-1 cell suspension was adjusted to a concentration of 1×10^6^ cells/ml, and 100 ng/ml of phorbose-12-myristose-13-acetate (PMA) was added for 6 h to induce them differentiation into M0. Following successful induction of M0, IFN-γ (20 ng/mL) and LPS (100 ng/mL) were added and incubated for 48 h to polarize the macrophages into M1. Similarly, IL-4 (20 ng/mL) and IL-13 (20 ng/mL) were added to M0, and cultured for 48 h to polarize them into M2.

Short hairpin RNAs (shRNAs), plasmids, and corresponding negative controls were purchased from Genepharma (Shanghai, China). These compounds were transported into corresponding cells using Lipofectamine 3000 (Invitrogen, USA) following the manufacturer's instructions.

### RNA extraction and quantitative reverse transcription-polymerase chain reaction (qRT-PCR)

Total RNA was extracted with TRIzol (Invitrogen, USA) and was qualified by agarose gel electrophoresis. Total RNA was reversely transcribed into cDNAs with a reverse transcription cDNA Kit (Thermo Fisher, USA). Next, qRT-PCR was conducted with a Real-Time PCR Kit (Takara, Japan) and the ABI 7500 system (Applied Biosystems, USA). GAPDH was used as the internal control, and the 2^-ΔΔCt^ method was used to calculate gene fold changes (FCs) 20. The primers used are shown in **Table S1**.

### Western blot

Total proteins were isolated from cells and tissues using the Whole Cell Lysis Assay Kit (Keygen Biotech, China), and their concentration was determined using the BCA Protein Quantitation Assay Kit (Keygen Biotech). Proteins were separated by 12% SDS-PAGE gel and transferred onto hydrophobic polyvinylidene fluoride (PVDF) membranes. After blocking with 5% skim milk, the membranes were incubated in the primary antibody overnight. After two hours of incubation with secondary antibodies, the membranes were exposed to an imaging machine (Tanon 5200, China) 21. The antibodies used are shown in **Table S2**.

### Transwell assay

Transwell assays were conducted with transwell insert chambers (Corning, USA). For migration assays, cells were digested with trypsin and resuspended with serum-free DMEM when they were in the logarithmic growth phase. Then, 50,000 cells were added to the upper chamber, and 500 μL of 10% FBS-containing DMEM were added to the lower chamber. After incubating chambers for 48 h, cells were fixed in anhydrous ethanol for 5 min. After wiping the upper chambers with cotton swabs, the remaining cells were stained with 1% crystal violet, captured, and counted. Unlike migration assays, the upper chambers used in invasion assays were pre-laid with 1:8 diluted Matrigel (Coring, USA) 22.

### Flow cytometry

To assess the polarization of macrophages, indicated cells were digested by pancreatic enzymes and collected by centrifugation. After being suspended and washed three times with phosphate-buffered saline (PBS, Gibco, USA), cells were incubated with antibodies for 20 min following the manufacturer's instructions. The cells incubated in the antibodies were then collected by centrifugation and washed with PBS. Finally, the fluorescence was measured on the Agilent Navocyte and analyzed with FlowJo. The antibodies used in this assay were CD68 (BioLegend, USA) and CD163 (BioLegend, USA).

### Isolation and validation of EVs

EV-free FBS used was purchased from Sciencell (USA). The media from cells cultured with EV-free FBS (10%) for 48 h was harvested. After being filtered using a 0.22 μm cellulose acetate filter, the harvested medium was centrifuged at 4 °C for 15 mins at 300 *g*, 4 °C for 20 min at 2,000 *g*, and 4 °C for 30 min at 16,500 *g*. Then, the supernatant was loaded onto a qEV column iZon (Cambridge, USA), and fractions were collected following the manufacturer's instructions. Finally, protein concentration was measured by BCA protein quantitation assay (Keygen Biotech), size distribution by nanoparticle tracking analysis (NTA) with Nanosight NS50 (Malvern Instruments, UK), and morphology by transmission electron microscopy (TME; Hitachi, Japan). The EVs markers (CD36, CD81 and Calnexin) were verified by Western blot assay.

### Identification of EV endocytosis

Isolated EVs were labeled with PKH67 using PKH67 Green Fluorescent Cell Linker Kit (Solarbio, China). Next, labeled EVs were added into the medium to culture glioblastoma cells for 6 h in a 37°C, 5% CO_2_ incubator. Then, the cultured cells were fixed with paraformaldehyde (4%), and the nucleus was stained with DAPI for 5 min. The EV endocytosis was observed with confocal microscopy (Carl Zeiss, Germany).

### RNA-sequence (RNA-seq) and analysis

First, rRNAs were removed from total RNAs by Ribo-Zero rRNA Removal Kits (Illumina, USA). The sequenced library was constructed with a Stranded Total RNA Library Prep Kit (Illumina, USA). Quality control and quantification of the library were performed using BioAnalyzer 2100 (Agilent, USA). According to Illumina sequencing, the 10 pM library was denatured into single-stranded DNA molecules, captured on Illumina flowcell, and amplified into clusters *in situ*. The 150-cycle sequencing was performed in the PE mode in Illumina Novaseq 6000. Double-ended reads were harvested after sequencing. High-quality reads were compared to the reference genome/transcriptome using STAR software (v2.5.1b), and circRNAs were detected and identified using DCC software (v0.4.4). Identified circRNAs were annotated using the CircBase database and Circ2Traits. The edgeR software (v3.16.5) was used for data standardization and differentially expressed circRNAs screening.

### Biotin-labeled RNA pull-down

Biotin-labeled circ_0003137 and corresponding negative control probes were synthesized by Genepharma. RNA pull-down was conducted with EZ-Magna ChIRP RNA Interactome Kit (Millipore) following the manufacturer's instructions. The RNA-protein complex was divided into two parts, one for purifying RNA and the other for Western blot identification. The enrichment of circ_0003137 in purified RNA was evaluated by qRT-PCR, and target protein PTBP1 levels were evaluated by Western blot.

### RNA immunoprecipitation

RNA immunoprecipitation was conducted using Magna RIP^TM^ RNA Binding Protein Immunoprecipitation Kit (Millipore, USA) following the manufacturer's instructions. Magnetic beads were incubated with antibodies for 25 min at room temperature. Cell lysate and pre-treated magnetic beads were incubated overnight at 4 °C. Next, RNA-protein complexes were digested by proteinase K digestion buffer for 1 h at 55 °C. Enriched RNAs were isolated using a special mixture (phenol: chloroform: isoamyl alcohol = 125:24:1) and detected by qRT-PCR.

### Immunofluorescence (IF) and hematoxylin-eosin (H&E) staining

Tissues were embedded in paraffin and made into slices (4 μm). For the IF assay, tissue sections were dewaxed after being placed at room temperature for 1 h, and 3% hydrogen peroxide was added to tissue sections and incubated at room temperature for 10 min. Subsequently, goat serum sealer was added to the sections and left at room temperature for 20 min. Then, primary antibodies were added to sections overnight. The secondary antibody was added to sections and incubated for 2 h. After 10 min of DAB treatment, sections were washed with PBS three times, stained with hematoxylin for 2 min, and washed with PBS three times. Finally, sections were sealed and captured.

For H&E, paraffin was removed from sections with xylene, and sections were soaked in varying alcohol concentrations (from high to low). Pre-treated sections were placed in a hematoxylin water solution for staining for 5 min, then decolorized with ammonia, alcohol, and distilled water successively. Finally, decolorized sections were sealed with resin and photographed 23.

### Actinomycin D assay

Glioma cells (5 × 10^6^) were treated with actinomycin D (2 μg/ml, AAT Bioquest, USA) for 0, 4, 8, and 12 h, respectively. Next, the total RNA of these cells was extracted, and the expression of PLOD3 was detected by qRT-PCR.

### Nanoplatform synthesis

Nanocarrier construction was described in our previous study 24. The 5-Benzyl-L-glutamate-N-carboxy-anhydride (Glu-NCA), DOTAP, and Met were purchased from Aladdin (Shanghai, China). RGD-PEG2000-DSPE and PLGA (50:50, Mw = 7000-17000) were purchased from Xi'an Ruixi Biological Technology (Xi'an, China). D-Luciferin potassium salt was purchased from Beyotime (Shanghai, China). Sh-circ_0003137, sh-NC, FAM labeled sh-circ_0003137, and FAM labeled sh-NC were purchased from Genepharma. To construct the RDPP(Met) complex, RGD-PEG2000-DSPE, DOTAP, PLGA, and PEG2000-Poly (met) were first added to anhydrous ethanol in a 1:4:1:8 proportion [PLGA:DOTAP:RGD-PEG2000-DSPE:PEG2000-Poly(met)] and slowly added to ultra-pure water for 40 min for full mixing. To remove the ethanol, the RDPP(Met) complex was purified with a dialysis membrane (7000 Dal. Next, shRNA was added to RDPP(Met) complex at a nitrogen/phosphate (N/P) ratio of 2.5. Finally, the nanoplatform was characterized using TEM and nanoparticle tracking analysis (NTA).

### Establishment of blood-brain barrier (BBB) model *in vitro*

An *in vitro* BBB model was established using co-culture insert chambers (0.4 μm, Millipore, Merck, Germany) according to our previous study 24. bEnd.3 cells (5 × 10^4^) were seeded in the upper chambers, and 800 μl culture medium was added to the lower chambers. Next, the trans-endothelial electrical resistance (TEER) of the monolayer bEnd.3 was detected using the Artemis System (Locsense, Netherlands). A TEER value exceeding 200 was considered indicative of a functional BBB model for further research.

### Animal study

Male blab/c mice, four weeks old (ten per group), were purchased from Gempharmatech (Nanjing, China). LN229-Luci cells at logarithmic growth were resuspended with serum-free DMEM, and the cell density was adjusted to 1 x10^7^/ml. Mice were anesthetized by 0.3% pentobarbital sodium via intraperitoneal injection, and LN229 (50,000) was injected into the right caudate nucleus of mice with stereotaxic help. Then, a living imaging system was used to monitor tumor growth at indicated days.

For nanoplatform-dependent treatment assay, glioblastoma orthotopic xenograft models were constructed following the above method. At 12, 14, and 16 d after LN229 transplantation, sh-circ_0003137-loaded nanoplatform was injected into mice via tail vein and imaged at the indicated time. All animal procedures complied with the Institutional Animal Care and Use Committee (IACUC) guidelines and were approved by the ethics committee of Nanjing Medical University (No.2110017).

### Statistical analysis

Data were analyzed by GraphPad Prism (version 9.3) and are shown as means ± standard deviations (SD). For comparison between two groups, a *t*-test was performed. Survival analysis was conducted using the Kaplan-Meier method and evaluated by the log-rank test. * *p* < 0.05 and ** *p* < 0.01 between two groups were considered statistically significant.

## Results

### M2-like TAMs are elevated in glioblastomas and associated with prognosis

**Figure 1:**
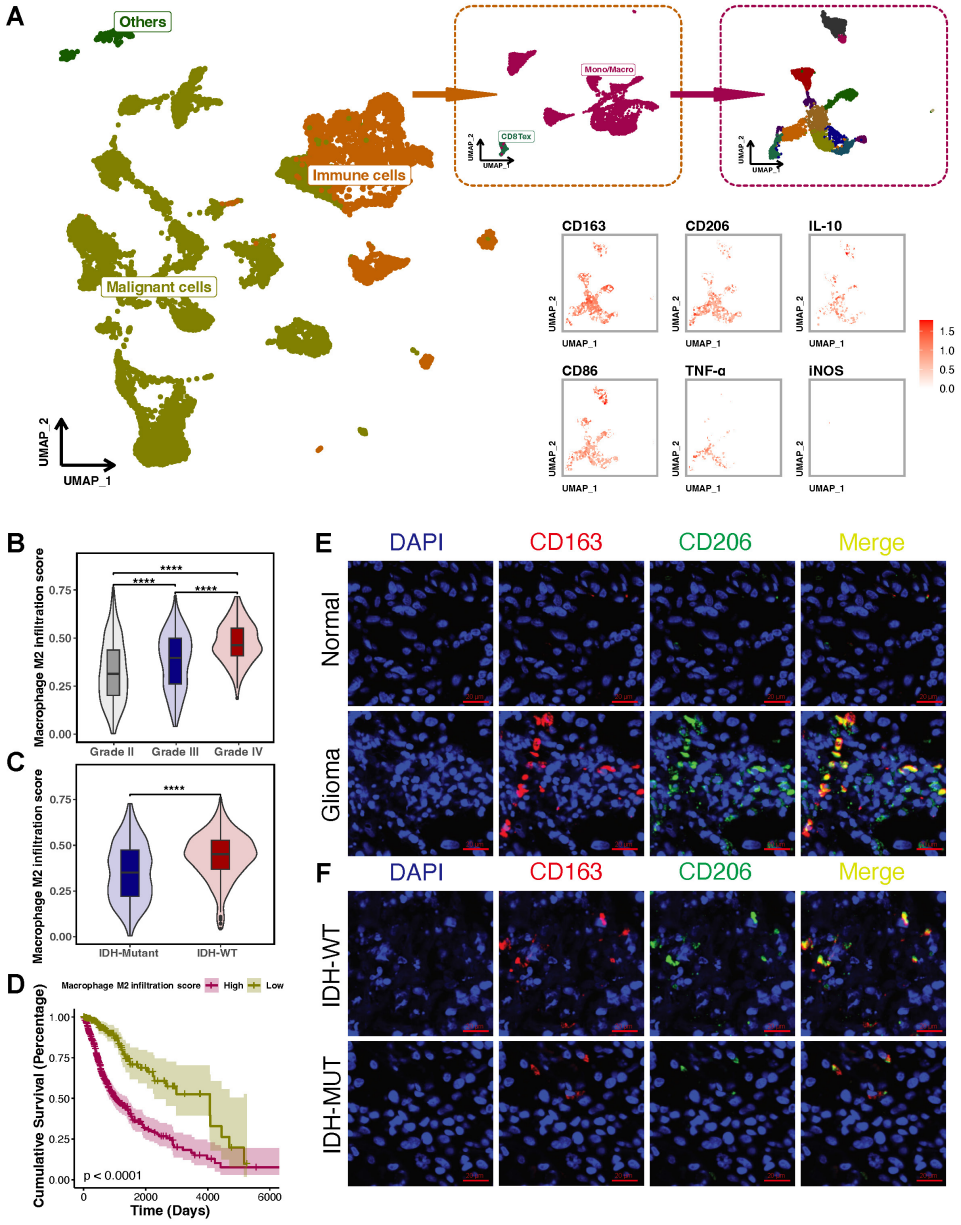
** M2-like TAMs are elevated in glioblastomas and associated with patients**'** prognosis. (A)** Single-cell RNA-seq data from GEO depicted a subcluster of significantly abundant TAMs, primarily polarized toward the M2 phenotype. The individuals within the dashed box on the left are all from the immune cells cluster (orange). The individuals within the dashed box on the right are all from the Mono/Macro cluster (burgundy). **(B)** Single-sample gene set enrichment analysis of M2-like TAMs signature genes in TCGA glioblastoma dataset showed that the M2-like TAMs signature score increased with higher grades. **(C)** M2-like TAMs signature score is higher in IDH-WT than in IDH-Mutant. **(D)** The Kaplan-Meier survival analysis of the M2-like TAMs signature score showed that a higher score indicated a poorer prognosis. **(E)** IF staining of CD163 and CD206 showed that more M2-like TAMs infiltrate in glioblastoma tissue than normal brain tissue. **(F)** IF staining of CD163 and CD206 showed that compared with IDH-WT, fewer M2-like TAMs infiltrate in IDH-Mutant glioblastoma tissue.

Single-cell RNA-seq glioblastoma data from the GEO database (GSE131928) was analyzed to investigate the role of M2-like TAMs in glioblastomas. TAMs accounted for about 29.27% of total cells, mainly M2-like TAMs (CD163, CD206 and IL-10 positive cells) (**Figure 1A**). To clarify M2-like TAMs infiltration, we compared the scores of M2-like TAMs across glioblastoma specimens collected in TCGA. M2-like TAMs infiltration increased with increasing glioblastoma grades and was more pronounced in IDH-wild type (WT) than in IDH-mutant glioblastomas (**Figures 1B and C**). Next, the Kaplan-Meier analysis was used to determine the relationship between the M2-like TAMs signature and the clinical prognosis of glioblastomas. Glioblastoma patients with higher M2-like TAMs infiltration had a lower survival rate (**Figure 1D**). Furthermore, IF staining for CD163 and CD206 was conducted in glioblastoma specimens to confirm the above results. Compared to normal brain tissues, glioblastoma tissues contained more CD163/CD206 positive cells (**Figure 1E**). Similarly, more CD163^+^/CD206^+^ cells were detected in IDH-WT than in IDH-mutant glioblastomas (**Figure 1F**). Therefore, M2-like TAMs are elevated in glioblastomas and negatively associated with their prognosis.

### Hypoxia promotes the transformation of THP-1-derived macrophages into the M2 subtype

**Figure 2:**
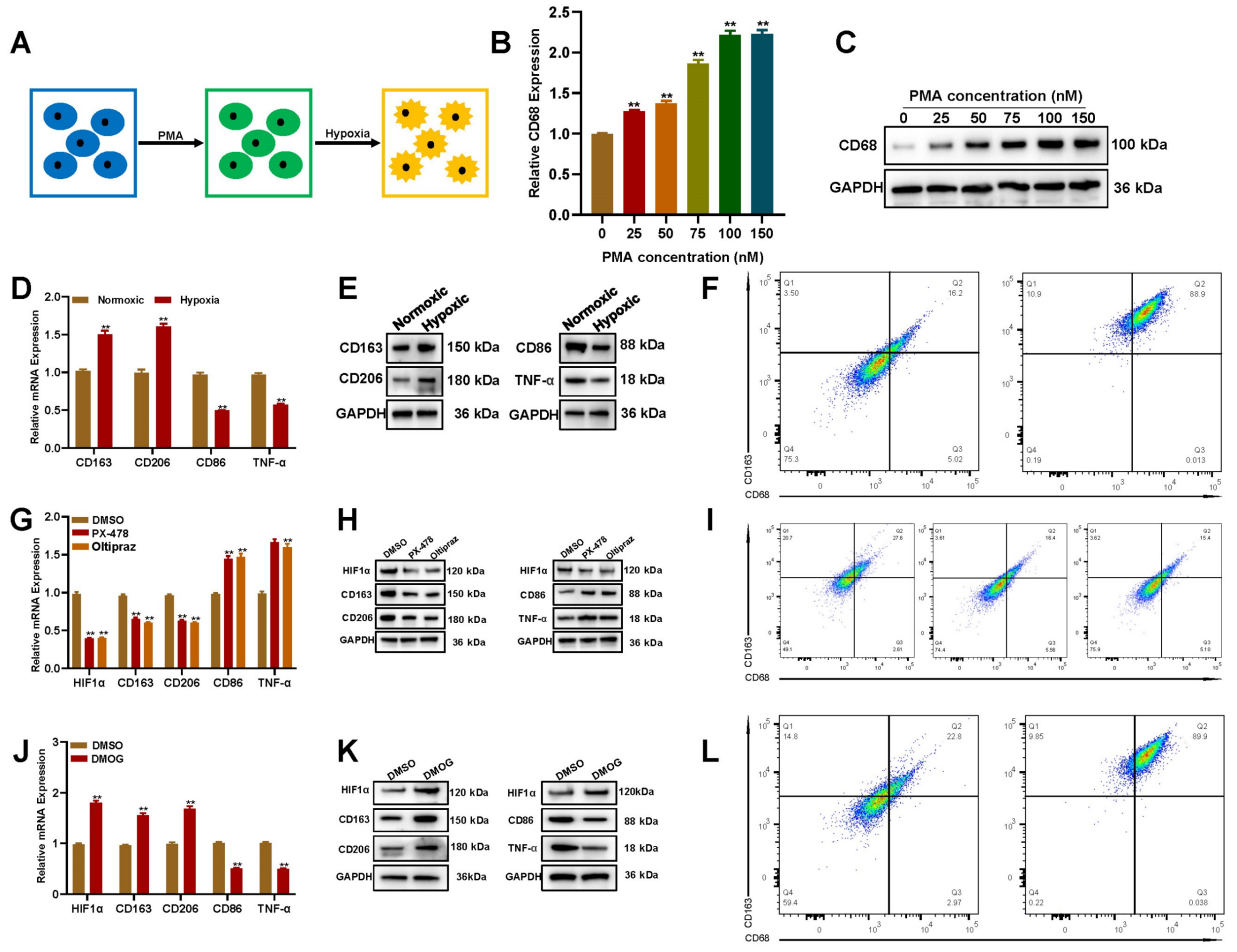
** Hypoxia promotes the transformation of THP-1-derived macrophages into the M2 subtype. (A)** Schematic illustration for the induction of M2 macrophages from THP-1 cells. **(B-C)** PMA induces the conversion of THP-1 to M0 in a dose-dependent manner by qRT-PCR and Western blot. **(D-F)** Oxygen deprivation promotes M0 polarization toward M2 by qRT-PCR, Western blot, and flow cytometry. **(G-I)** Inhibiting HIF1α decreased the M0 polarization toward M2 driven by oxygen deprivation. **(J-L)** HIF1α stabilization promoted M0 polarization toward M2 driven by oxygen deprivation.

Hypoxia is an important characteristic of the glioblastoma microenvironment, facilitating glioblastoma development by changing the biological phenotypes of parenchymal and stromal cells 18, 25. To explore whether hypoxia can promote macrophage polarization towards M2-like TAMs, THP-1 was differentiated to M0 by phorbol 12-myristate 13-acetate (PMA), then cultured under hypoxia (**Figure 2A**). The qRT-PCR and Western blot assay showed that after being treated with PMA for 72 h, CD68 (M0 marker) was upregulated in THP-1 cells (**Figures 2B and C**). Next, PMA-induced M0 cells were cultured under hypoxia (1% O_2_) for 48 h, and the levels of M1/M2-associated markers were detected by qRT-PCR and Western blot. Hypoxia not only upregulated CD163 and CD206 but also downregulated CD86 and TNF-α (**Figures 2D and E**), indicating that hypoxia could promote M0 polarization toward the M2 phenotype. The M0-to-M2 transition under hypoxia was also verified by flow cytometry (**Figure 2F**).

Hypoxia-inducible factor (HIF), especially HIF1α, is critical to the hypoxia response signal transduction 26. Thus, to explore whether HIF1α was involved in hypoxia-induced M0-to-M2 transition, inhibitors targeting HIF1α were used to treat with M0 cells cultured under hypoxia. The qRT-PCR and Western blot assay showed that CD163 and CD206 were downregulated in HIF1α inhibitor (PX-478/Oltipraz) treated cells (**Figures 2G and H**). The flow cytometry analysis showed that the population of CD163^+^/CD206^+^ cells decreased in HIF1α inhibitor treated cells (**Figure 2I**). Consistently, the HIF1α stabilizer, dimethyloxalylglycine (DMOG), significantly enhanced CD163 and CD206 expression under normoxia after 48 h (**Figures 2J-L**). These findings suggested that hypoxia promotes the polarization of THP-1-derived M0 into M2 macrophages in a HIF1α dependent manner.

### M2-like TAMs facilitate migration and invasion of glioblastomas via EV transport

**Figure 3:**
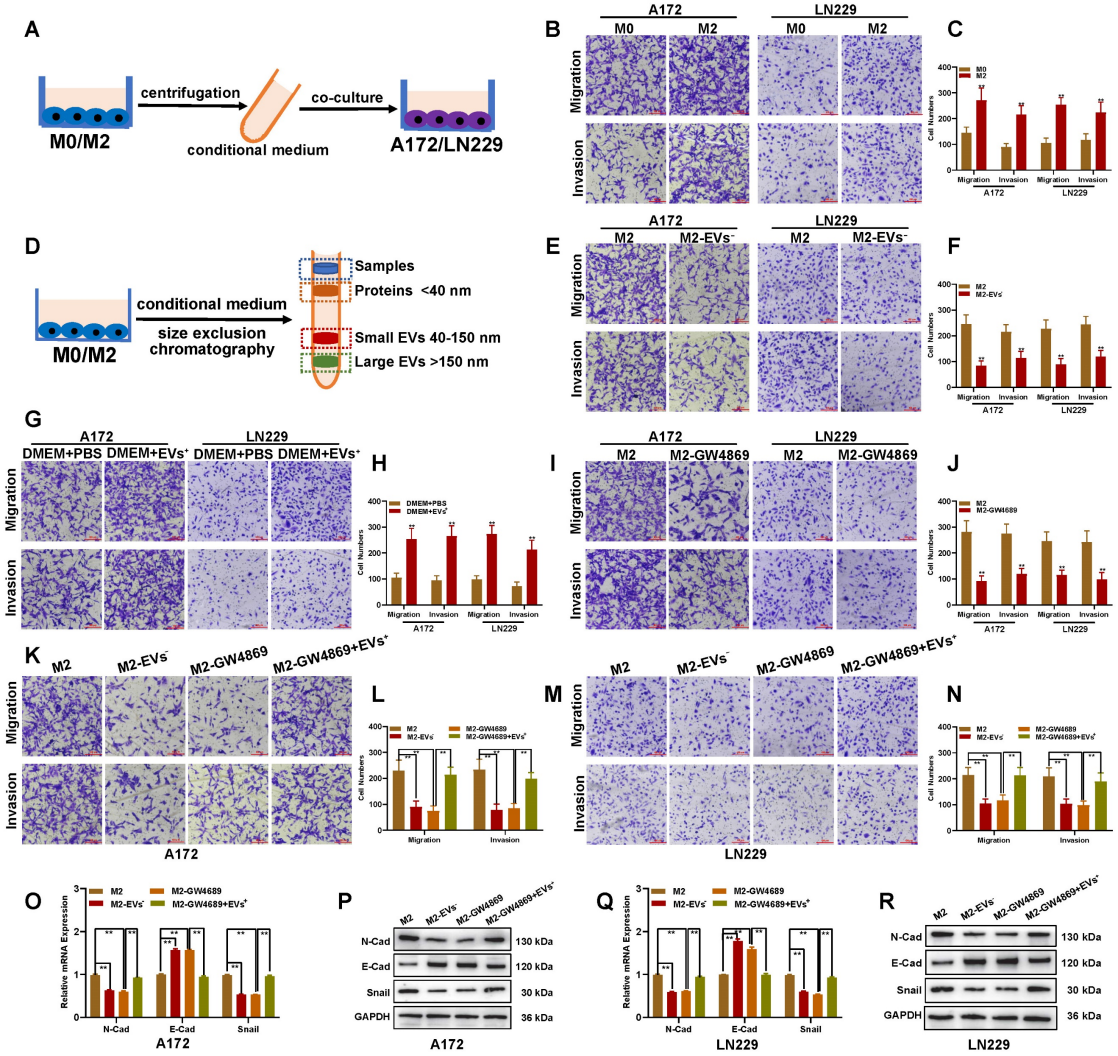
** M2 macrophages facilitate glioblastoma migration and invasion by transporting extracellular vesicles. (A)** Schematic illustration for the co-culture of glioblastoma cells with conditional medium from M2-like TAMs. **(B-C)** The migration and invasion ability of glioblastoma cells was enhanced when co-cultured with M2-like TAMs-derived conditional medium. **(D)** Schematic illustration for extracellular vesicles (EVs) or protein separated from M2-like TAMs-derived conditional medium. **(E-F)** The migration and invasion ability of glioblastoma cells treated with M2-like TAMs-derived conditional medium with or without EVs by Transwell assay. **(G-H)** The migration and invasion ability of glioblastoma cells treated with PBS or M2-like TAMs-derived EVs was measured by Transwell assay. **(I-J)** The EVs inhibitor restrained the migration and invasion ability of glioblastoma cells treated with the M2-like TAMs-derived conditional medium. **(K-N)** The role of GW4869 on glioblastoma migration and invasion could be reversed by M2-like TAMs-derived EVs. **(O-R)** EMT markers' level in indicated groups by qRT-PCR and Western blot.

Moreover, M2-like TAMs could sustain the tumor-promoting microenvironment through interaction with other tumor cells 27. To investigate whether hypoxia-induced M2-like TAMs facilitate glioblastoma progress, glioblastoma cells were cultured with the conditional medium from M0 and hypoxia-induced M2-like TAMs (THP-1-derived) (**Figure 3A**). The transwell assay demonstrated that hypoxia-induced M2-derived medium significantly enhanced glioblastoma cells' migration and invasion abilities (**Figures 3B and C**). Since M2 did not directly contact glioblastoma cells, we hypothesized that M2 macrophages facilitated the migration and invasion ability of glioblastoma cells through EVs. EV fractions from the conditional medium were isolated by size exclusion chromatography (**Figure 3D**). Culturing glioblastoma cells with an M2-derived conditional medium without EVs reduced their migration and invasion abilities (**Figure 3E-3F**). Meanwhile, when glioblastoma cells were cultured with fresh DMEM containing EVs from M2 macrophages, their migration and invasion abilities were significantly enhanced (**Figures 3G and H**). To further clarify the role of M2 EVs in promoting glioblastoma migration and invasion, GW4869 was used to inhibit EVs release. The transwell assay showed that GW4869 suppressed the migration and invasion of glioblastoma cells co-cultured with the M2-derived conditional medium (**Figures 3I-J**). Moreover, the inhibition effect of GW4869 was reversed by additional EVs derived from M2 macrophages (**Figures 3K-N**). The qRT-PCR and Western blot assays indicated that the EMT marker N-Cad and Snail were downregulated in co-cultured glioblastoma cells, while E-Cad was upregulated when M2-derived EVs were excluded or inhibited by GW4689. At the same time, this change could be reversed by additional M2-derived EVs (**Figures 3O-R**).

To verify whether M2 macrophages can promote migration and invasion of glioblastoma cells through EVs in glioblastoma tissues, CD11b and CD163 were used as markers to separate M2 from glioblastoma tissues by fluorescence-activated cell sorting. The transwell assay indicated that glioblastoma cells co-cultured with M2 (CD11b^+^/CD163^+^)-derived conditional medium in which EVs were excluded or inhibited by GW4689 showed decreased migration and invasion ability, and the decrease could be restrained by M2 (CD11b^+^/CD163^+^) derived EVs (**Figures S1A-D**). Expression changes of EMT-associated markers in co-cultured glioblastoma cells were similar to THP-1-derived M2 macrophages (**Figures S1E-H**). These results suggested that M2-like TAMs facilitate the migration and invasion of glioblastomas via EVs transport.

### M2-like TAMs-derived extracellular vesicles promote the migration and invasion of glioblastomas by circ_0003137 carrying

Furthermore, circRNAs play diverse roles in tumorigenesis and can be transported by EVs to recipient cells 28. Thus, to explore whether circRNAs were transported in M2-derived EVs and promoted the migration and invasion of glioblastoma cells, EVs from M0 and M2 macrophages were isolated. TEM and NTA showed that EVs were typical rounded particles with approximately 60-100 nm diameter (**Figures S2A-C**). The presence of EVs was evaluated using CD63, CD81, calnexin and TSG101 levels by Western blot (**Figure S2D**). To explore whether EVs could be phagocyted by glioblastoma cells, EVs were labeled with PKH67 and added to the culture medium of glioblastoma cells. Fluorescence imaging suggested that glioblastoma cells could internalize EVs after 2 h of co-culture (**Figure S2E**).

**Figure 4:**
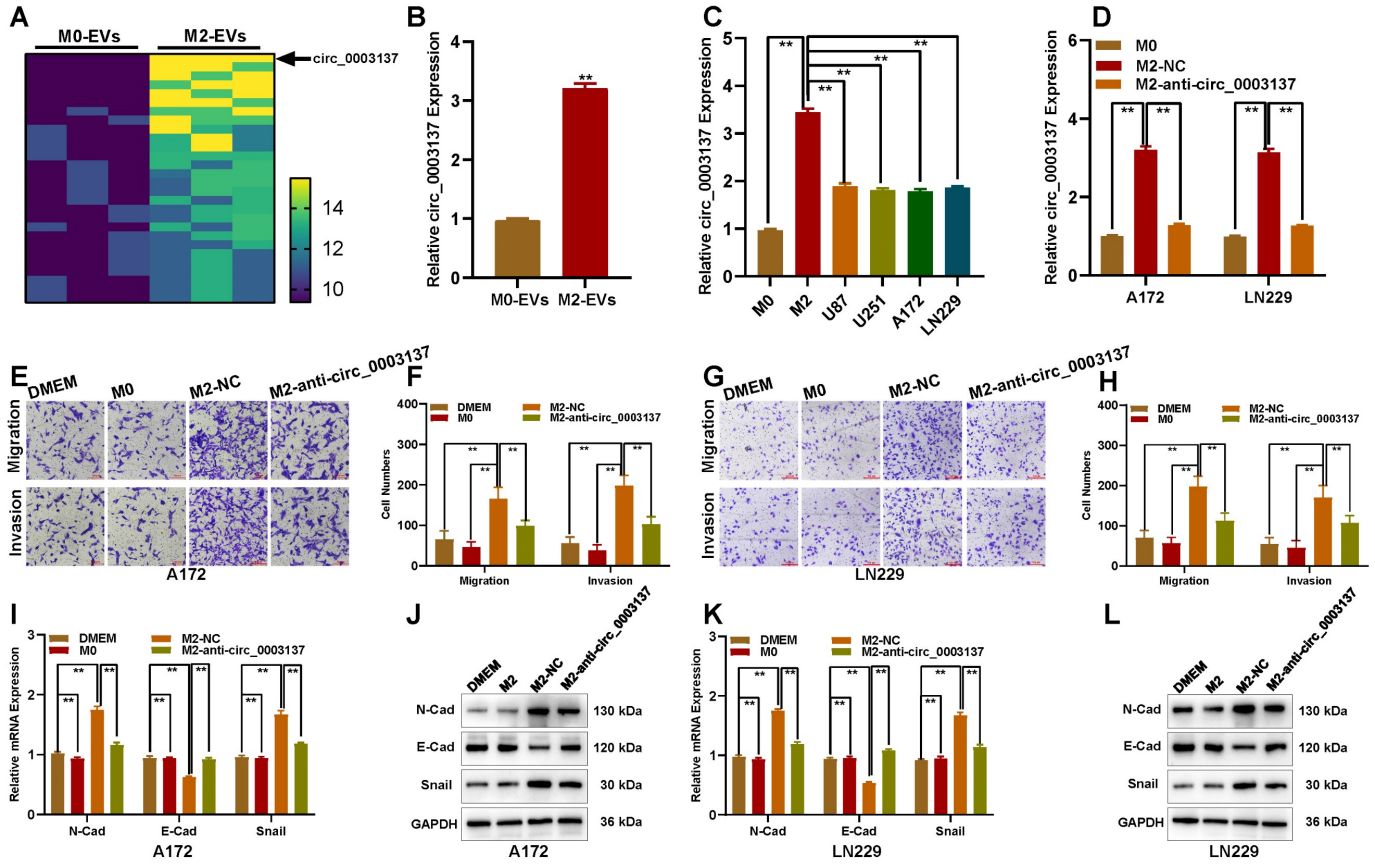
** M2-like TAMs-derived extracellular vesicles promote the migration and invasion of glioblastomas by carrying circ_0003137. (A)** Twenty-eight circRNAs were upregulated in M2-EVs compared to M0-EVs. **(B)** Expression of circ_0003137 in M0-EVs and M2-EVs by qRT-PCR. **(C)** Expression of circ_0003137 in M0, M2, and glioblastoma cells. **(D)** Expression of circ_0003137 in glioblastoma cells co-cultured with indicated cells. **(E-H)** The migration and invasion ability of glioblastoma cells co-cultured with indicated cells. **(I-L)** EMT markers' level in glioblastoma cells co-cultured with indicated cells.

To explore key circRNAs, high-throughput sequencing based on the Illumina platform was used to detect differentially expressed circRNAs in M0 and M2-derived EVs. With |log_2_FC| ≥ 2 and *p* < 0.05 as screening conditions, 28 circRNAs were highly expressed in M2 compared with M0-derived EVs, and circ_0003137 was the most upregulated (**Figure 4A**). Circ_0003137 is derived from CTNNB1; its genomic loci are shown in **Figure S3A**. RNase R enzyme could degrade linear CTNNB1 (parent gene of circ_0003137) but did not affect circ_0003137 (**Figures S3B and C**), confirming the circular structure of circ_0003137. The Kaplan-Meier analysis demonstrated that higher circ_0003137 was related to poorer survival of glioblastoma patients (**Figure S3D**). Moreover, the Transwell assay showed that circ_0003137 promoted the migration and invasion of glioblastoma cells related to EMT process (**Figure S4**). The qRT-PCR indicated that the circ_0003137 content in M2-derived EVs was three times higher than in M0-derived EVs (**Figure 4B**). Otherwise, circ_0003137 was higher in M2 than in glioblastoma cells (**Figure 4C**). To confirm the circ_0003137 transport by EVs, M2 macrophages were transfected with anti-circ_0003137 and negative control, respectively. The qRT-PCR showed that circ_0003137 expression was downregulated in glioblastoma cells co-cultured with anti-circ_0003137-transfected M2 macrophages (**Figure 4D**). The Transwell assay indicated that the migration and invasion ability of glioblastoma cells co-cultured with anti-circ_0003137-transfected M2 macrophages was suppressed (**Figures 4E-H**). Compared to the negative control, N-Cad and Snail were downregulated and E-Cad was upregulated in glioblastoma cells co-cultured with anti-circ_0003137-transfected M2 macrophages (**Figures 4I-L**).

**Figure 5:**
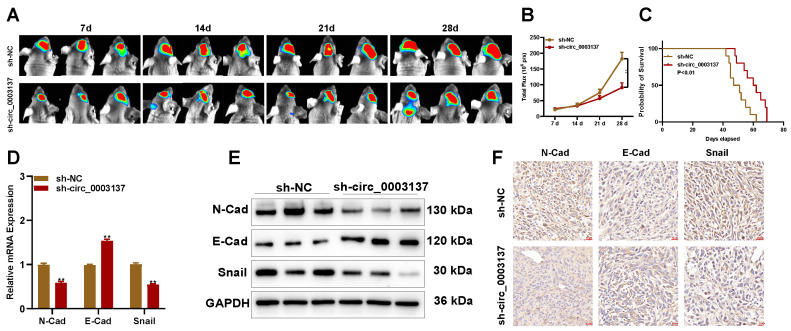
** Downregulation of circ_0003137 inhibits glioblastoma progression *in vivo*. (A-B)** Bioluminescent images of intracerebral tumors formed by LN229 transfected with sh-NC or sh-circ_0003137. **(C)** Kaplan-Meier survival analysis of mice bearing intracerebral tumors. **(D-F)** EMT markers' level in intracerebral tumors formed by LN229 were detected by qRT-PCR, Western blot, and IHC.

To evaluate the role of circ_0003137 *in vivo*, an animal model was constructed with LN229-luci transfected with sh-circ_0003137 or negative control. The *in vivo* imaging showed that the tumors formed by LN229-luci transfected with sh-circ_0003137 slowed glioblastoma progression (**Figures 5A and B**). Kaplan-Meier analysis demonstrated that the mice bearing tumors in the sh-circ_0003137 group had better survival (**Figure 5C**). The levels of EMT-associated proteins in tumors were detected by qRT-PCR, Western blot, and IHC. N-Cad and Snail were downregulated and E-Cad was upregulated in tumors formed by sh-circ_0003137-transfected LN229 (**Figures 5D-F**). These findings suggested that M2-like TAMs-derived EVs promote the migration and invasion of glioblastomas by carrying circ_0003137.

### Circ_0003137 promotes the migration and invasion of glioblastomas by recruiting PTBP1

**Figure 6:**
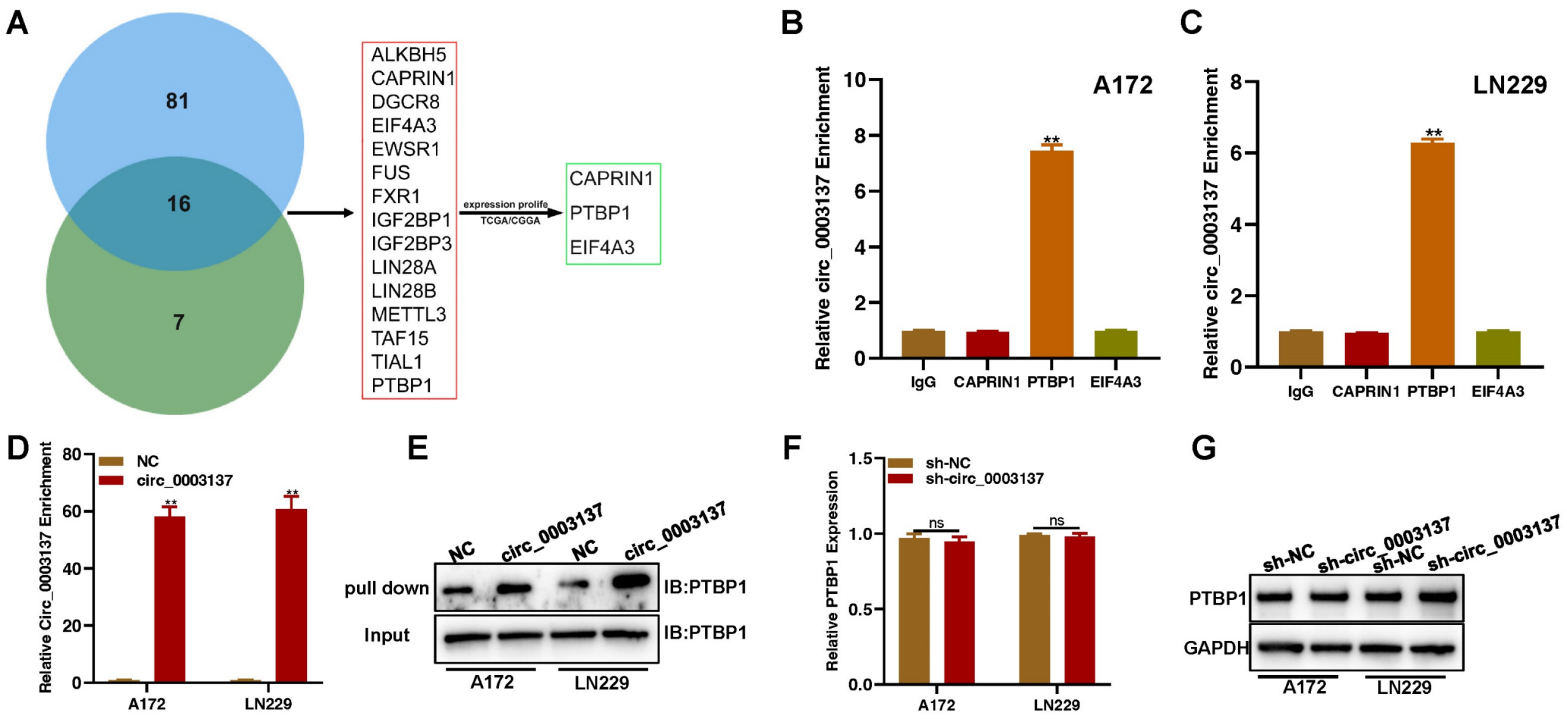
** Circ_0003137 promotes the migration and invasion of glioblastoma by recruiting PTBP1. (A)** Schematic illustration for preliminary screening of circ_0003137 downstream molecules based on TCGA, CGGA, starBase, and circular RNA interactome. **(B-C)** RIP-qPCR analysis showed the enrichment of circ_0003137 on indicated RBPs. **(D-E)** Western blot showed that PTBP1 was pulled down using the circ_0003137-specific probe. **(F-G)** PTBP1 expression in circ_0003137-silenced glioblastoma cells by qRT-PCR and Western blot.

Existing evidence has shown that circRNAs function mainly by regulating the expression of its parent gene, sponging miRNAs, or targeting RNA binding proteins (RBPs) 29. The qRT-PCR and Western blot assays showed that circ_0003137 did not affect CTNNB1 levels (**Figures S5A and B**). To investigate whether circ_0003137 functions as a miRNA sponge, the RIP assay was carried out with an antibody against argonaute (AGO2). The circ_0003137 enrichment did not differ between AGO2 and control (IgG) groups (**Figure S5C**), which suggested that circ_0003137 could not be a miRNA sponge in glioblastoma cells. Next, starBase and circinteractome were used to screen RBPs that might be targeted by circ_0003137. The Venn diagram showed that the two databases shared 16 RBPs (**Figure 6A**). The expression of 16 RBPs in glioblastomas based on TCGA and CCGA is shown in **Figure S6**, and CAPRIN1, PTBP1, and EIF4A3 were selected for further analysis. To determine the RBPs targeted by circ_0003137 in glioblastoma cells, the RIP assay was conducted with antibodies against CAPRIN1, PTBP1, and EIF4A3. Only the PTBP1 immunoprecipitate was enriched for circ_0003137 (**Figures 6B and C**). Moreover, the RNA pull-down was conducted with biotin-labeled circ_0003137. Compared to controls, circ_0003137 and PTBP1 were significantly enriched in the circ_0003137 group (**Figures 6D and E**). In contrast, the qRT-PCR and Western blot assay showed that circ_0003137 silence did not affect PTBP1 levels (**Figures 6F and G**). These findings suggested that circ_0003137 physically binds to PTBP1.

Furthermore, the relationship between PTBP1 expression and glioblastoma clinical features was evaluated based on TCGA and CCGA. PTBP1 was closely related to patients' age, glioblastoma grade, IDH mutation, 1p/19q deletion, MGMT methylation, and survival time (**Figure S7**). Then. PTBP1 expression was assessed in glioblastoma tissues and cell lines. PTBP1 was upregulated in glioblastoma tissues and cell lines (**Figures S8A-D**). Next, PTBP1-silenced glioblastoma cells were constructed to evaluate the function of PTBP1 on glioblastoma migration and invasion (**Figure S8E-S8F**). The Transwell assay showed that PTBP1 downregulation impaired the migration and invasion of glioblastoma cells (**Figure S8G-S8J**). Moreover, EMT-associated proteins were also involved in the regulatory effect of PTBP1 (**Figures S8K and L**). These results demonstrated that circ_0003137 affects glioblastoma migration and invasion by physically binding to PTBP1.

### PLOD3 is involved in circ_0003137/PTBP1-induced glioblastoma migration and invasion

**Figure 7:**
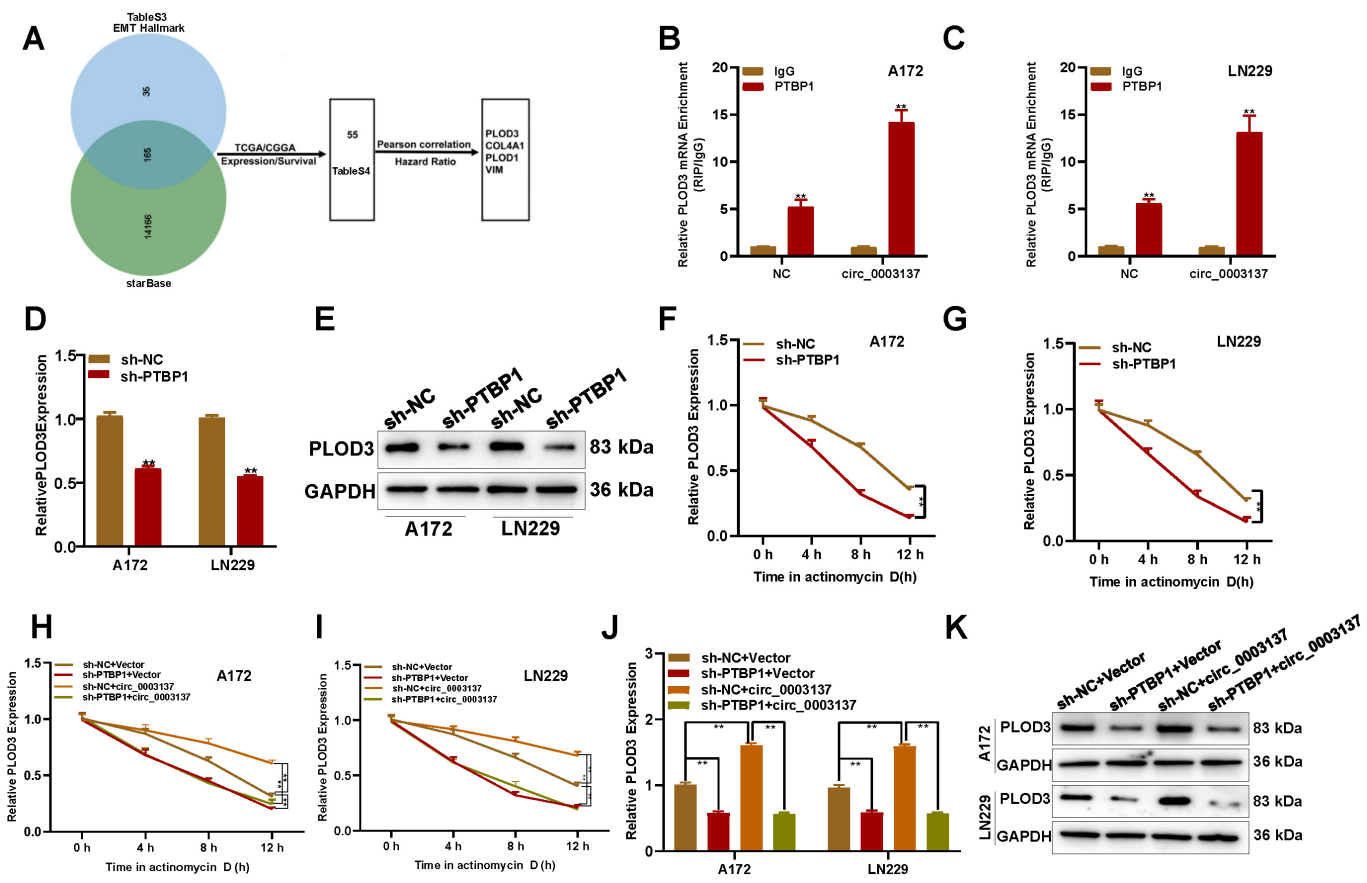
** PLOD3 is involved in the circ_0003137/PTBP1-induced migration and invasion of glioblastomas. (A)** Schematic illustration for the preliminary screen downstream of PTBP1. **(B-C)** RIP-qPCR analysis showed the enrichment of PLOD3 on PTBP1 in circ_0003137-overexpressed glioblastoma cells. **(D-E)** PLOD3 expression in PTBP1-silenced glioblastoma cells by qRT-PCR and Western blot. **(F-G)** Effects of PTBP1 silencing on the stability of PLOD3 mRNA in glioblastoma cells treated evaluated using actinomycin D. **(H-I)** PLOD3 expression in indicated glioblastoma cells by qRT-PCR and Western blot. **(J-K)** The stability of PLOD3 mRNA in indicated glioblastoma cells was evaluated using actinomycin D.

To determine the downstream effects of PTBP1, starBase was used to search potential targets of PTBP1, and 14318 candidates were selected. However, only 165 were related to the EMT (**Figure 7A, Table S3**). According to Pearson correlation and hazard ratio, PLOD3, COL4A1, PLOD1, and VIM were selected for further study (**Figure S9A-S9H, Table S4**). The RIP-PCR assays showed that only PLOD3 could be enriched by PTBP1 in circ_0003137-overexpressed glioblastoma cells (**Figures 7B-C and S9I-K**). Furthermore, PTBP1 downregulation inhibited PLOD3 expression (**Figures 7D-E**). These data indicated that PLOD3 might be downstream of the circ_0003137/PTBP1 axis.

To investigate the role of PLOD3 in glioblastomas, the relationship between PLOD3 and glioblastoma clinical features was also studied based on TCGA and CCGA. PLOD3 was closely related to patients' age, glioblastoma grade, IDH mutation, 1p/19q deletion, MGMT methylation, and survival time (**Figure S10**). Also, PLOD3 was upregulated in glioblastoma tissues and cell lines (**Figure S11A-D**). Then, PLOD3-silenced glioblastoma cells were constructed to assess its effects on glioblastoma migration and invasion (**Figure S11E and F**). The Transwell assay showed that PLOD3 downregulation could suppress the migration and invasion of glioblastoma cells (**Figures S11G-J**). Moreover, EMT-associated proteins were also involved in the regulatory effects of PLOD3 (**Figure S11K-S11L**).

Next, the underlying mechanisms of PLOD3 downregulation regulated by PTBP1 silence were explored. Actinomycin D treatment showed that PTBP1 silence could decrease PLOD3 mRNA levels by enhancing its degradation (**Figures 7F and G**). Moreover, the qRT-PCR and Western blot assays showed that PTBP1 absence could abolish the promotion effect of circ_0003137 on PLOD3 expression (**Figures 7H and I**). Similarly, circ_0003137 could not change the half-life of PLOD3 in PTBP1-deleted glioblastoma cells (**Figure 7J-7K**). Based on these findings, rescue experiments were conducted. The Transwell assay revealed that, when PTBP1 was absent, circ_0003137 overexpression did not affect the migration and invasion of glioblastoma cells (**Figure S12A-S12D**). Finally, EMT-associated protein levels were detected by qRT-PCR and Western blot. Without PTBP1, upregulation of circ_0003137 did not affect N-Cad, E-Cad, and Snail (**Figure S12E-S12H**). Altogether, the migration and invasion of glioblastoma cells could be facilitated by circ_0003137 via the PTBP1/PLOD3 axis.

### The sh-circ_0003137-loaded nanoplatform inhibits the EMT of glioblastomas *in vivo*

Our previous work established a nanoplatform capable of delivering shRNAs based on liposomes, where the specific synthesis process and characterization of the relevant properties are described 24. To evaluate the inhibitory effect of circ_0003137 silence on the malignant progression of glioblastomas *in vivo*, the sh-circ_0003137-loaded nanoplatform was injected via the tail vein into mice. The TEM and NTA showed that the sh-circ_0003137-loaded nanoplatform was well dispersed with a narrow size distribution, and their average diameters were about 100 nm (**Figure S13A**). To explore whether the sh-circ_0003137-loaded nanoplatform was effectively taken up by cells, the nanoplatform was labeled by FAM, then added to the culture supernatant of glioblastoma cells. The fluorescence microscope tracer indicated that glioblastoma cells could effectively internalize the nanoplatform (**Figures S13B and C**). Transwell assays showed that the sh-circ_0003137-loaded nanoplatform could significantly inhibit migration and invasion of glioblastoma cells (**Figures S13D-G**). Furthermore, EMT-associated protein levels in glioblastoma cells were also regulated by the nanoplatform (**Figures S13H-K**). These results demonstrated that the sh-circ_0003137-loaded nanoplatform was stable and could be taken up by glioblastoma cells, affecting their behavior.

The blood-brain barrier (BBB) is a great obstacle in drug treatments for CNS diseases. To verify whether the sh-circ_0003137-loaded nanoplatform could penetrate the BBB and reach the CNS after injection through the tail vein, an *in vitro* BBB model was constructed with bEnd.3 and A172 cells 30 (**Figure S14A**). Compared to sh-circ_0003137 alone, the cumulative transport ratio of the sh-circ_0003137-loaded nanoplatform was higher, suggesting efficient BBB permeability (**Figure S14B**). Additionally, the fluorescence microscope tracer detected the sh-circ_0003137 taken up by A172 in lower chambers, and compared to the control, the sh-circ_0003137-loaded nanoplatform was endocytic by A172 more effectively (**Figure S14C**). Therefore, the sh-circ_0003137-loaded nanoplatform could penetrate the BBB after tail vein injection, providing a basis for *in vivo* glioblastoma treatment.

**Figure 8:**
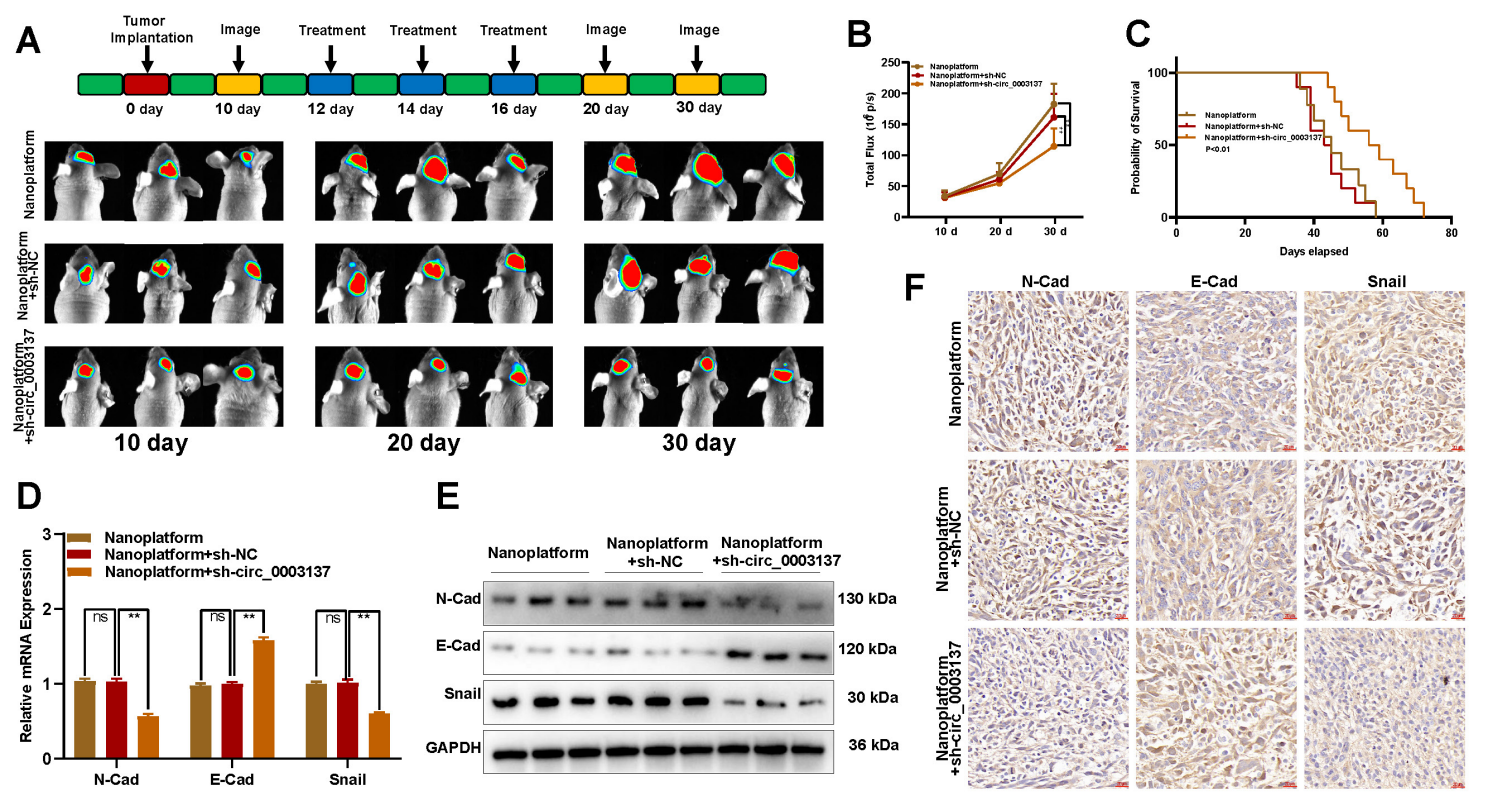
** The sh-circ_0003137-loaded nanoplatform inhibited the EMT of glioblastomas *in vivo*. (A**) Schematic illustration for *in vivo* experiments. The LN229-luci-tumor bearing model was established and treated with nanoplatform, nanoplatform + sh-NC, or nanoplatform + sh-circ_0003137. **(B)** Bioluminescent measurement of intracerebral tumors treated with nanoplatform, nanoplatform + sh-NC, or nanoplatform + sh-circ_0003137. **(C)** Kaplan-Meier survival analysis of mice bearing intracerebral tumors. **(D-F)** EMT markers' level in intracerebral tumors treated with nanoplatform, nanoplatform + sh-NC, or nanoplatform + sh-circ_0003137 by qRT-PCR, Western blot, and IHC.

Intracranial glioblastoma models were constructed to test the therapeutic effect of the sh-circ_0003137-loaded nanoplatform on glioblastoma *in vivo*. On the 12, 14, and 16th days after intracranial inoculation of LN229, the sh-circ_0003137-loaded nanoplatform was injected through the tail vein. The *in vivo* imaging indicated that this nanoplatform could suppress malignant progression (**Figures 8A and B**). The Kaplan-Meier analysis demonstrated that mice treated with the sh-circ_0003137-loaded nanoplatform had better survival (**Figure 8C**). Additionally, EMT-associated protein levels in tumors were detected by qRT-PCR, Western blot, and IHC. N-Cad and Snail were downregulated, while E-Cad was upregulated in tumors of mice treated with the sh-circ_0003137-loaded nanoplatform (**Figures 8D-F**). Overall, these results showed that the sh-circ_0003137-loaded nanoplatform could inhibit glioblastoma progression in an EMT-dependent manner.

Based on these findings, the fluorescence microscope tracer showed the distribution of sh-circ_0003137 in mice's brains, demonstrating the ability of the nanoplatform to penetrate the BBB (**Figure S14D**). Also, the major organs of mice were excised, and the H&E staining indicated no tissue damage or morphological changes (**Figure S14E**). These results suggested that the sh-circ_0003137-loaded nanoplatform could inhibit the progression of glioblastoma safely and effectively.

## Discussion

Glioblastomas are characterized by high proliferation and invasive growth and account for about 70% of CNS malignant tumors, comprising a serious challenge in neurosurgery 31. The strong migration and invasion ability of glioblastoma cells is one of the reasons for the poor prognosis of glioblastoma patients. EMT is a key process for glioblastoma cells to acquire highly migratory and invasive phenotypes. Based on these foundations, understanding the molecular regulator network of glioblastomas and the EMT is essential for developing glioblastoma-targeted therapy strategies.

TAMs are one of the most abundant inflammatory cells in the TME, accounting for about 25-45% of glioblastoma tissues 32. They are mainly derived from monocytes in peripheral blood circulation and macrophages of inherent embryonic origin in tissues, such as microglia cells in normal brain tissues. In recent years, increasing studies have shown that TAMs are important in promoting the whole cascade process of tumor metastasis, providing therapeutic targets for inhibiting tumor progression 33. At primary tumor sites, TAMs can promote migration and invasion of tumor cells through various mechanisms, increasing tumor cells' metastasis potential 34. TAMs at distant metastatic sites are also involved in the progression of tumor metastasis. They cannot only migrate to the metastatic site before the arrival of tumor cells to reconstruct the TME of the metastatic site but also directly act on tumor cells to promote their exudation, colonization, and growth at the metastatic site 35. TAMs play a very important role in glioblastoma progression. An experiment simulating glioblastoma growth reported that, in the 2D co-cultured assay, TAMs showed phagocytic ability and produced a killing effect on glioblastoma cells. However, in 3D co-cultured assays, TAMs lost phagocytic activity and changed from a pro-inflammatory and active form to an immunosuppressive state, contributing to glioblastoma growth, migration, and invasion 36, 37. It is generally believed that the immune function of TAMs will change from pro-inflammatory to anti-inflammatory under the influence of surrounding tumor tissues when recruited into glioblastoma tissues and promote glioblastoma development. Herein, the single-cell sequencing data showed that TAMs accounted for 29.27% of glioblastoma tissues and were associated with glioblastoma grade, IDH state, and patient prognosis. Therefore, studying TAMs polarization and related driving factors is of great value.

Influenced by complex cellular components and cytokines in the TME, TAMs have significant heterogeneity and complexity. Currently, the most classical classification divides TAMs into two subtypes: M1-like TAMs, involved in the Th1 response, and M2-like TAMs, involved in the Th2 response. In benign tumors, TAMs show the M1 phenotype, with pro-inflammatory activity to kill microorganisms and promote tumor lysis 38. TAMs exhibit the M2 phenotype in malignant tumors and promote tumor progress mainly by secreting immunosuppressive factors. As the most common malignant tumor of adult CNS, the infiltrating TAMs in glioblastoma tissues have M2-like characteristics. The polarization of macrophages towards M1 or M2 phenotypes is regulated by the tumor and the TME. A study reported that ovarian epithelial cancer cells could promote M1 polarization toward M2 39. In our previous study, glioblastoma cells with high DHX9 expression could recruit macrophages and trigger their polarization toward the M2 phenotype 2. However, the underlying molecular mechanisms by which the glioblastoma TME promotes the polarization of macrophages towards the M2 phenotype remain unclear.

Hypoxia is one of the important characteristics of the TME, affecting the behavior of tumor cells, and is currently considered a key driving force for tumor progression 40. Clinical data have indicated that hypoxia is associated with tumor metastasis and poor prognosis in patients 41. In Glioblastoma, hypoxia can induce tumor parenchymal cells to polarize macrophages towards the M2 phenotype 42. Nevertheless, the direct effects of hypoxia on macrophage polarization have never been reported. Here, THP-1 cells were induced into M0 macrophages by PMA, then cultured under hypoxic conditions. M0 cells presented M2 characteristics after 48 h of hypoxic culture. HIF1α is the main effector of hypoxic stimulation. Using the HIF1α inhibitor (PX-478/Oltipraz) and stabilizer (DMOG), we demonstrated that the inhibition of HIF1α weakened the ability to induce M0 to M2, while overexpression enhanced it. These findings suggested that hypoxia could polarize M0 toward M2 in an HIF1α dependent manner in glioblastomas.

Most studies insist that M2-like TAMs achieve their tumor-promoting effect by releasing various cytokines to reconstruct the TME. In the present study, M2 cells were co-cultured with glioblastoma cells, and M2 cells could promote the EMT of glioblastoma cells. To explore why M2-like TAMs promote the EMT in glioblastoma cells, the supernatant of M2-like TAMs (derived from both THP-1 and glioma tissues) was collected through centrifugation. Through the use of GW4869 and transcriptomic sequencing, we confirmed that M2-like TAMs promote EMT by transferring EVs to glioblastoma cells, leading to circ_0003137 upregulation. As an important regulatory molecule, circRNAs are closely related to glioblastoma EMT. Wang *et al.* reported that circXPO1 promotes glioblastoma migration and invasion via miR-7 sponging 43. Cao *et al.* showed that circNDC80 promotes the migration and invasion of glioblastoma cells by ECE1 regulation 44. In our study, with the help of various bioinformatics and molecular biology experiments, we found that circ_0003137 regulated glioblastoma EMT via the PTBP1/PLOD3 signaling axis. PTBP1 has been reported to be dysregulated in several caners, including gastric, bladder, and colorectal cancers 45. Studies also indicated that PTBP1 was upregulated in glioma tissues, and promote the malignant phenotype of glioma cells 46, however, its regulatory network and potential treatments need further exploration. In our study, we showed that higher PTBP1 expression indicated poorer prognosis in glioma patients. Mechanism study indicated that PTBP1 was recruited by circ_003137, which was transported by EVs from Hypoxic induced M2-like TAMs. PLOD3, a membrane-bound homodimer enzyme, is involved in the biosynthesis and glycosylation of collagen. Increased collagen deposition and crosslinking enhance tumor cell migration and invasion. While PLOD3 is known to be highly expressed in various tumors 47, however, its role in the malignant progression of glioma has not been fully explored. This study found that PLOD3 is regulated by PTBP1. Bioinformatics analysis based on TCGA/CGGA and cell function experiments suggested that PLOD3 function as an oncogene in glioma progress. Furthermore, rescue experiments validated that PLOD3 is the functional target of circ_003137/PTBP1, thus regulating EMT of glioma cells.

The biggest obstacle to glioblastoma drug therapy is the BBB, which prevents drugs from reaching the CNS or reaching effective drug concentration. Many nanotechnology-based drug delivery systems have been conceived, and with the advantages of high drug load and long blood circulation time, nanotechnology-based drug delivery systems have become one of the most promising treatment methods for glioblastoma 48. Liposomes are tiny vesicles made of the same material as cell membranes wildly used in drug delivery because of their superior physicochemical properties and excellent biocompatibility 49. Liposomes are approved by the USA's Food and Drug Administration (FDA) for clinical use as nanoparticle systems. In our previous study, a nanoplatform based on liposomes was constructed their properties were demonstrated 24. In the present study, shRNA targeting circ_0003137 was encapsulated in this nanoplatform. TEM and Zetasizer NanoZS confirmed the stability of the sh-circ_0003137-loaded nanoplatform, and the cell experiments showed that it could be phagocytized by glioblastoma cells, inhibiting the EMT progression of glioblastoma cells. The animal experiments indicated that the nanoplatform could deliver sh-circ_0003137 to CNS and effectively inhibit glioblastoma EMT after being injected via the tail vein.

## Conclusions

**Figure 9:**
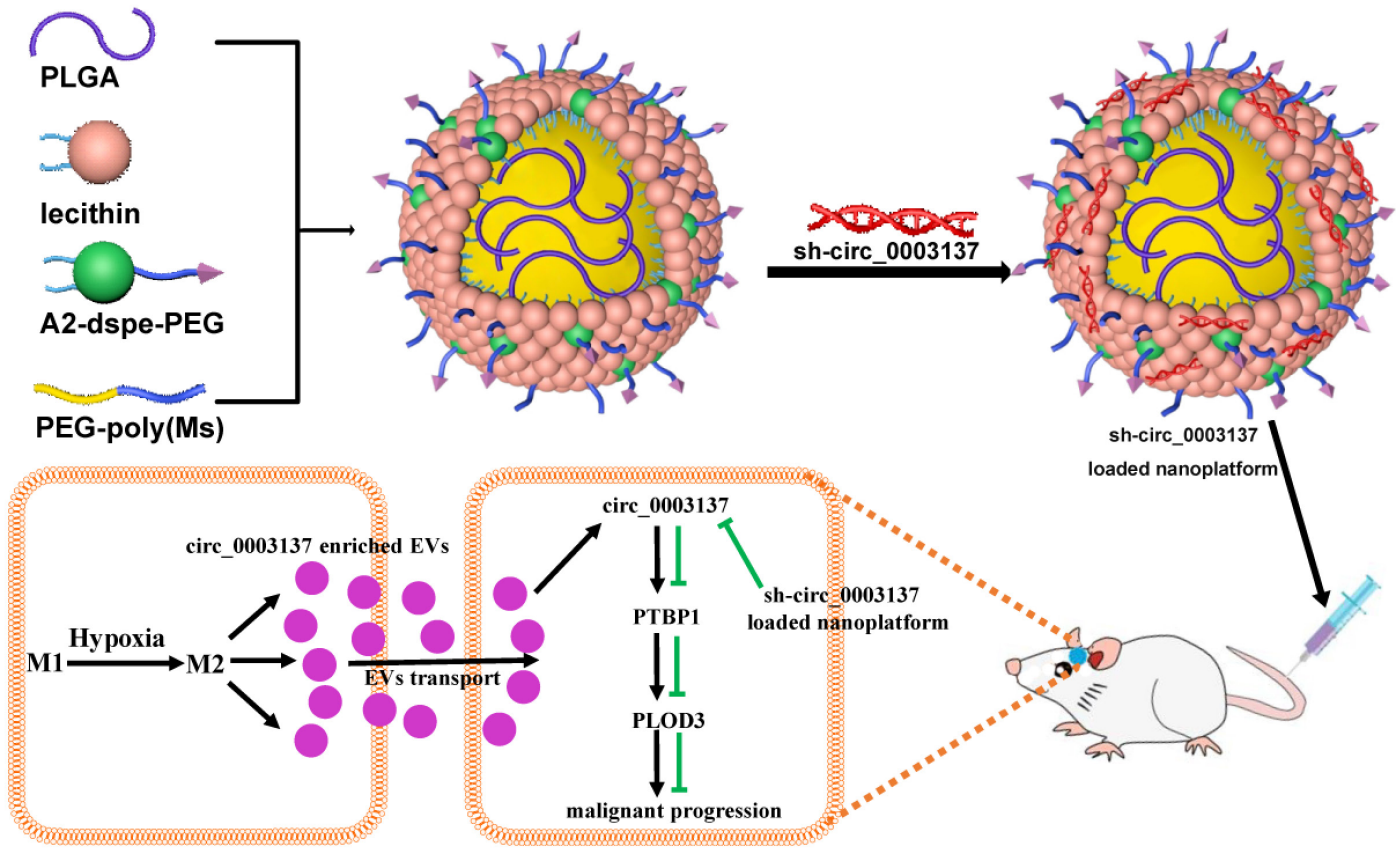
Working model of hypoxia-driven M2-polarized macrophages facilitate the epithelia-mesenchymal transition of glioblastoma via extracellular vesicles.

In conclusion, hypoxia could drive M0 polarization toward M2-like TAMs, which transport circ_0003137 to glioblastoma cells via EVs. Next, circ_0003117 promotes glioblastoma cell EMT by targeting the PTBP1/PLOD3 axis. Additionally, the sh-circ_0003137-loaded nanoplatform could deliver sh-circ_0003137 to CNS and inhibit the EMT of glioblastomas (**Figure 9**). Our findings suggested that circ_0003137 might be a novel target for glioblastoma therapy.

## Supplementary Material

Supplementary figures and tables.

## Abbreviations

TAMs: tumor-associated macrophages
EMT: epithelial-mesenchymal transition
EVs: extracellular vesicles
PTBP1: polypyrimidine tract binding protein 1
PLOD3: procollagen-lysine, 2-oxoglutarate 5-dioxygenase 3
BBB: blood-brain barrier
CNS: central nervous system
GBM: glioblastoma multiforme
GSCs: glioblastoma stem cells
TCGA: The Cancer Genome Atlas
NTA: nanoparticle tracking analysis
