# Supplementary material for: Hypoxia-driven M2-polarized macrophages facilitate the epithelial-mesenchymal transition of glioblastoma via extracellular vesicles
Source: Theranostics. 2024 Oct 7;14(16):6392–408. doi: 10.7150/thno.95766 (PMC11488104; doi:10.7150/thno.95766)
Supplement: Supplementary file 1 — Supplementary figures and tables. [file thnov14p6392s1.pdf]

Figure S1. M2 cells in glioblastomas tissues facilitate the migration and invasion of glioblastomas by transporting extracellular vesicles.
(A-D) The role of GW4869 on glioblastoma migration and invasion could be reversed by $CD11b^{+}/CD163^{+}$ cell-derived EVs.
(E-H) EMT markers' level in indicated groups by qRT-PCR and Western blot.

Figure S2. Characteristic of EVs isolated from M0 and M2 cells.
(A) Images of M0 and M2 derived EVs captured by transmission electron microscope.
(B-C) Size of EVs by nanoparticle tracking analysis.
(D) EVs markers detected by Western blot.
(E) EVs marked with PKH67 internalized by glioblastoma cells.

Figure S3. Characteristic of circ_0003137.
(A) Schematic illustration for the genomic loci of circ_0003137.
(B-C) Circ_0003137 RNA was resistant to RNase R.
(D) High circ_0003137 indicated poor survival of glioblastoma patients.

Figure S4. Role of circ_0003137 on glioblastoma EMT.
(A-D) The role of circ_0003137 on glioblastoma migration and invasion by Transwell assay.
(E-H) EMT markers' level in indicated groups by qRT-PCR and Western blot.

Figure S5. Role of circ_0003137 on parent gene and miRNAs.
(A-B) Role of circ_0003137 on CTNNB1 levels in glioblastoma cells by qRT-PCR and Western blot.
(C) RIP experiments were conducted using antibodies against AGO2 on extracts from glioblastoma cells.

Figure S6. Expression of 16 indicated RBPs in glioblastomas based on TCGA and CGGA.

Figure S7. Clinical features of PTBP1 in glioblastoma patients based on public databases.
(A-C) Clinical features of PTBP1 in glioblastoma patients based on TCGA.
(D-F) Clinical features of PTBP1 in glioblastoma patients based on CGGA.

Figure S8. Role of PTBP1 in glioblastoma progression.
(A-B) PTBP1 levels in glioblastoma tissues by qRT-PCR and Western blot.
(C-D) PTBP1 levels in glioblastoma cells by qRT-PCR and Western blot.
(E-F) PTBP1 levels by qRT-PCR analysis in glioblastoma cells transfected with sh-PTBP1 or corresponding negative control.
(G-J) Role of PTBP1 on glioblastoma migration and invasion by Transwell assay.
(K-L) EMT markers' level in indicated groups by qRT-PCR and Western blot.

Figure S9. Relationship between PTBP1 and COL4A1, PLOD1, VIM.
(A-D) Pearson correlation analysis showed a strong positive correlation between PTBP1 and PLOD3 based on TCGA.
(E-H) Pearson correlation analysis showed a strong positive correlation between PTBP1 and PLOD3 based on CGGA.
(I-K) RIP-qPCR analysis showed COL4A1, PLOD1, and VIM enrichment on PTBP1 in circ_0003137-overexpressed glioblastoma cells.

Figure S10. Clinical features of PLOD3 in glioblastoma patients.
(A-C) Clinical features of PLOD3 in glioblastoma patients based on TCGA.
(D-F) Clinical features of PLOD3 in glioblastoma patients based on CGGA.

Figure S11. Role of PLOD3 in glioblastoma progression.
(A-B) PLOD3 levels in glioblastoma tissues by qRT-PCR and Western blot.
(C-D) PLOD3 levels in glioblastoma cells by qRT-PCR and Western blot.
(E-F) PLOD3 expression by qRT-PCR in glioblastoma cells transfected with sh-PTBP1 or corresponding negative control.
(G-J) Role of PLOD3 on glioblastoma migration and invasion by Transwell assay.
(K-L) EMT markers' level in indicated groups by qRT-PCR and Western blot.

Figure S12. Role of PTBP1 in circ_0003137-regulated EMT progress.
(A-D) Migration and invasion ability of indicated glioblastoma cells by Transwell assay.
(E-H) EMT markers' level in indicated glioblastoma cells by qRT-PCR and Western blot.

Figure S13. Characteristics of the sh-circ_0003137-loaded nanoplatform system.
(A) Outline and size of the nanoplatform loaded with sh-NC or sh-circ_0003137 by TEM and Malvern Zetasizer NanoZS.
(B-C) Nanoplatform carried with sh-NC or sh-circ_0003137 can be phagocytosis by glioblastoma cells.
(D-G) Migration and invasion ability of glioblastoma cells treated with nanoplatform loaded with sh-NC or sh-circ_0003137 by Transwell assay.
(H-K) EMT markers' level in indicated glioblastoma cells by qRT-PCR and Western blot.

Figure S14. The sh-circ_0003137-loaded nanoplatform system could penetrate the BBB.
(A) Schematic illustration for the *in vitro* BBB model.
(B) Cumulative transport ratio of free FAM-sh-circ_0003137 and nanoplatform-carried FAM-sh-circ_0003137.
(C) Ability of glioblastoma cells to phagocytize shRNA in the BBB model by fluorescence microscopy.
(D) Images captured by fluorescence microscopy showed the distribution of FAM-shRNA in tumors formed by LN229.

(E) H&E staining of major organs extracted from mice treated with nanoplatform, nanoplatform + sh-NC, or nanoplatform + sh-circ_0003137.

Table S1 The primers used in this study.

Table S2 The antibodies used in Western blot assay.

Table S3 Eepithelial-mesenchymal transition hallmark genes set.

Table S4 The target genes of PTBP1 which screened according to their expression levels (based on TCGA).

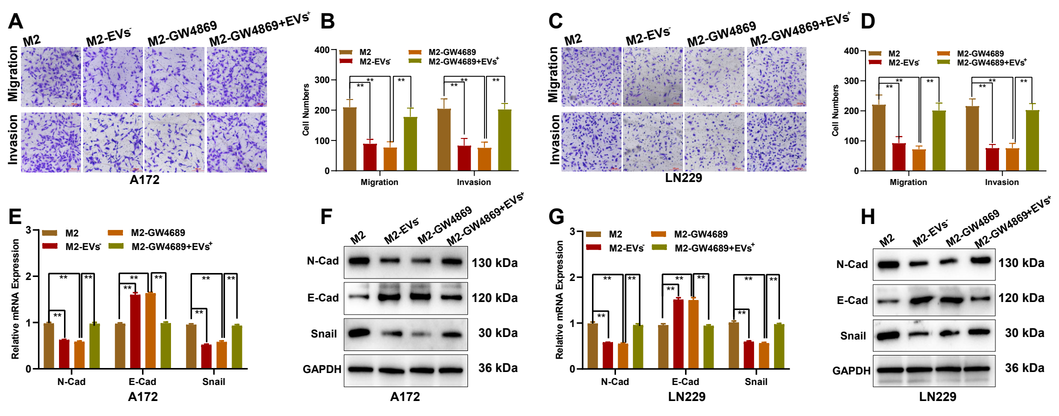

A
M2
M2-EVs-
M2-GW4869
M2-GW4869+EVs+
Migration
Invasion
A172
B
M2
M2-EVs-
M2-GW4689
M2-GW4689+EVs+
Cell Numbers
Migration
Invasion
C
LN229
D
E
Relative mRNA Expression
N-Cad
E-Cad
Snail
F
GAPDH
130 kDa
120 kDa
30 kDa
36 kDa
G
H

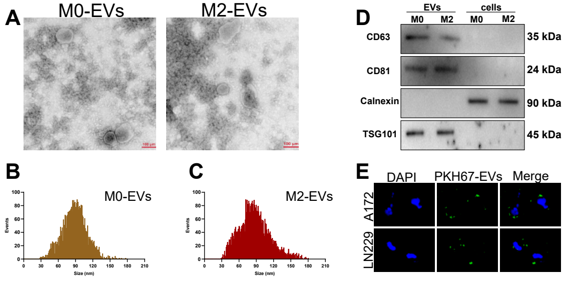
A
M0-EVs
M2-EVs
B
M0-EVs
Events
Size (nm)
C
M2-EVs
Events
Size (nm)
D
EVs
cells
M0
M2
M0
M2
CD63
35 kDa
CD81
24 kDa
Calnexin
90 kDa
TSG101
45 kDa
E
DAPI
PKH67-EVs
Merge
A172
LN229

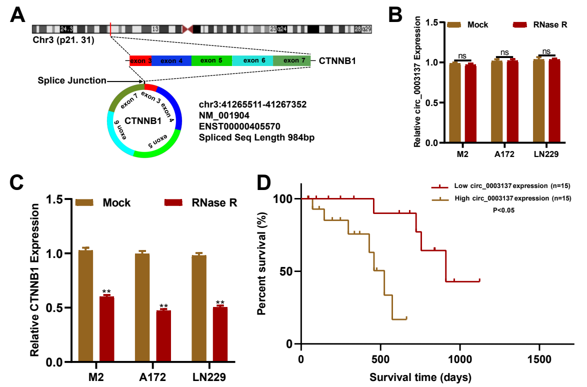
A
Chr3 (p21. 31)
exon 3
exon 4
exon 5
exon 6
exon 7
CTNNB1
Splice Junction
exon 7
exon 3
exon 4
exon 6
exon 5
CTNNB1
chr3:41265511-41267352
NM_001904
ENST00000405570
Spliced Seq Length 984bp
B
Mock
RNase R
Relative circ_0003137 Expression
ns
ns
ns
M2
A172
LN229
C
Mock
RNase R
Relative CTNNB1 Expression
**
**
**
M2
A172
LN229
D
Low circ_0003137 expression (n=15)
High circ_0003137 expression (n=15)
P<0.05
Percent survival (%)
Survival time (days)

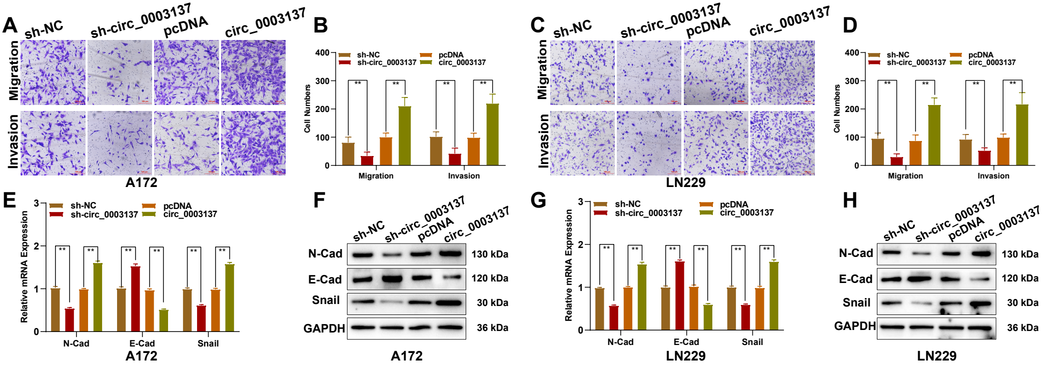

A
sh-NC
sh-circ_0003137
pcDNA
circ_0003137
Migration
Invasion
A172
B
Cell Numbers
sh-NC
sh-circ_0003137
pcDNA
circ_0003137
Migration
Invasion
C
LN229
D
E
Relative mRNA Expression
N-Cad
E-Cad
Snail
F
130 kDa
120 kDa
30 kDa
36 kDa
GAPDH
G
H

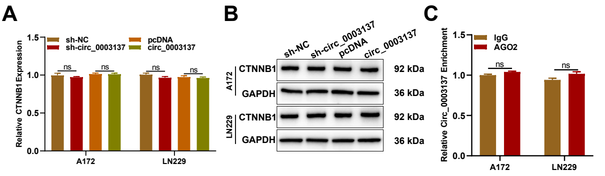
A
sh-NC
sh-circ_0003137
pcDNA
circ_0003137
Relative CTNNB1 Expression
1.5
1.0
0.5
0.0
ns
ns
ns
ns
A172
LN229
B
sh-NC
sh-circ_0003137
pcDNA
circ_0003137
A172
CTNNB1
GAPDH
LN229
CTNNB1
GAPDH
92 kDa
36 kDa
92 kDa
36 kDa
C
IgG
AGO2
Relative Circ_0003137 Enrichment
1.5
1.0
0.5
0.0
ns
ns
A172
LN229

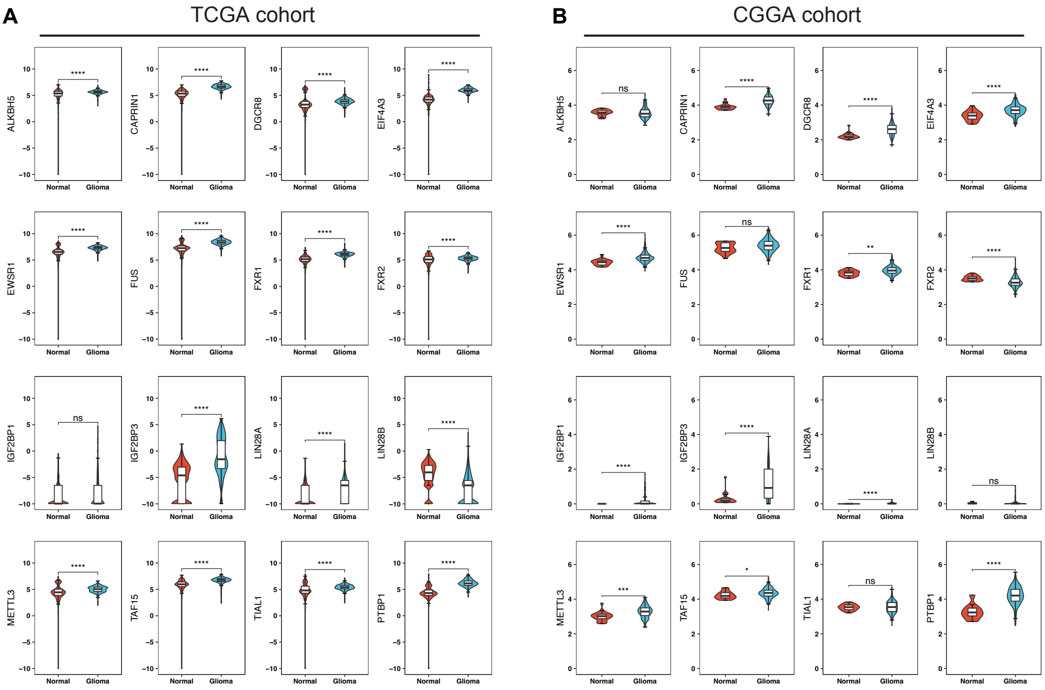
A
TCGA cohort
B
CGGA cohort
ALKBH5
CAPRIN1
DGCR8
EIF4A3
EWSR1
FUS
FXR1
FXR2
IGF2BP1
IGF2BP3
LIN28A
LIN28B
METTL3
TAF15
TIAL1
PTBP1
Normal
Glioma
****
***
**
*
ns

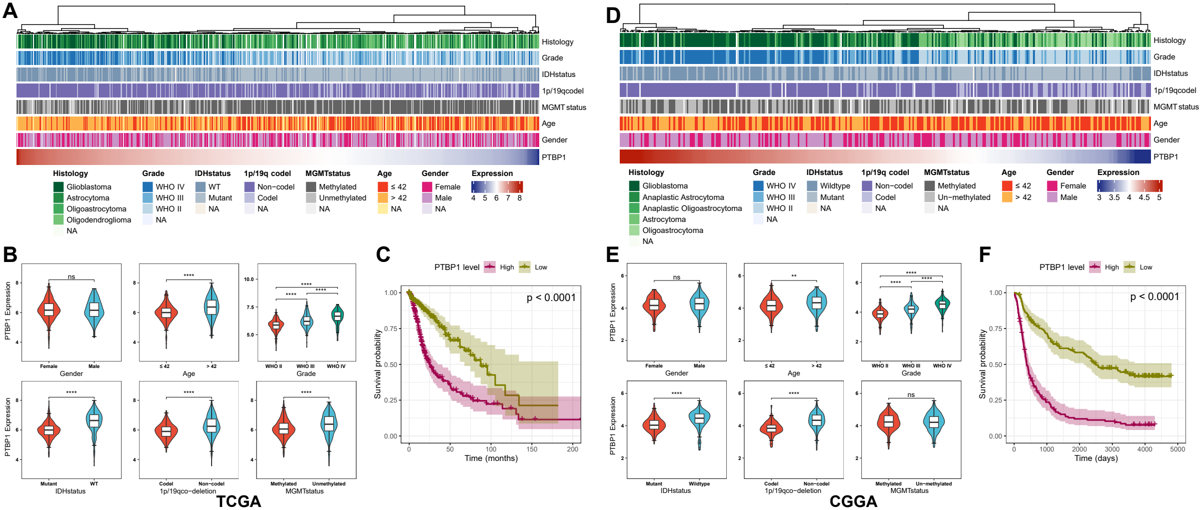
A
Histology
Grade
IDHstatus
1p/19qcodel
MGMT status
Age
Gender
PTBP1
Histology
Glioblastoma
Astrocytoma
Oligoastrocytoma
Oligodendroglioma
NA
Grade
WHO IV
WHO III
WHO II
NA
IDHstatus
WT
Mutant
NA
1p/19q codel
Non−codel
Codel
NA
MGMTstatus
Methylated
Unmethylated
NA
Age
≤ 42
> 42
NA
Gender
Female
Male
NA
Expression
4 5 6 7 8
D
Histology
Grade
IDHstatus
1p/19qcodel
MGMT status
Age
Gender
PTBP1
Histology
Glioblastoma
Anaplastic Astrocytoma
Anaplastic Oligoastrocytoma
Astrocytoma
Oligoastrocytoma
NA
Grade
WHO IV
WHO III
WHO II
NA
IDHstatus
Wildtype
Mutant
NA
1p/19q codel
Non−codel
Codel
NA
MGMTstatus
Methylated
Un−methylated
NA
Age
≤ 42
> 42
Gender
Female
Male
Expression
3 3.5 4 4.5 5
B
PTBP1 Expression
Gender
Female
Male
ns
Age
≤ 42
> 42
****
Grade
WHO II
WHO III
WHO IV
IDHstatus
Mutant
WT
1p/19qco−deletion
Codel
Non−codel
MGMTstatus
Methylated
Unmethylated
C
PTBP1 level
High
Low
p < 0.0001
Survival probability
Time (months)
TCGA
E
PTBP1 Expression
Gender
Female
Male
ns
Age
≤ 42
> 42
**
Grade
WHO II
WHO III
WHO IV
IDHstatus
Mutant
Wildtype
1p/19qco−deletion
Codel
Non−codel
MGMTstatus
Methylated
Un−methylated
F
PTBP1 level
High
Low
p < 0.0001
Survival probability
Time (days)
CGGA

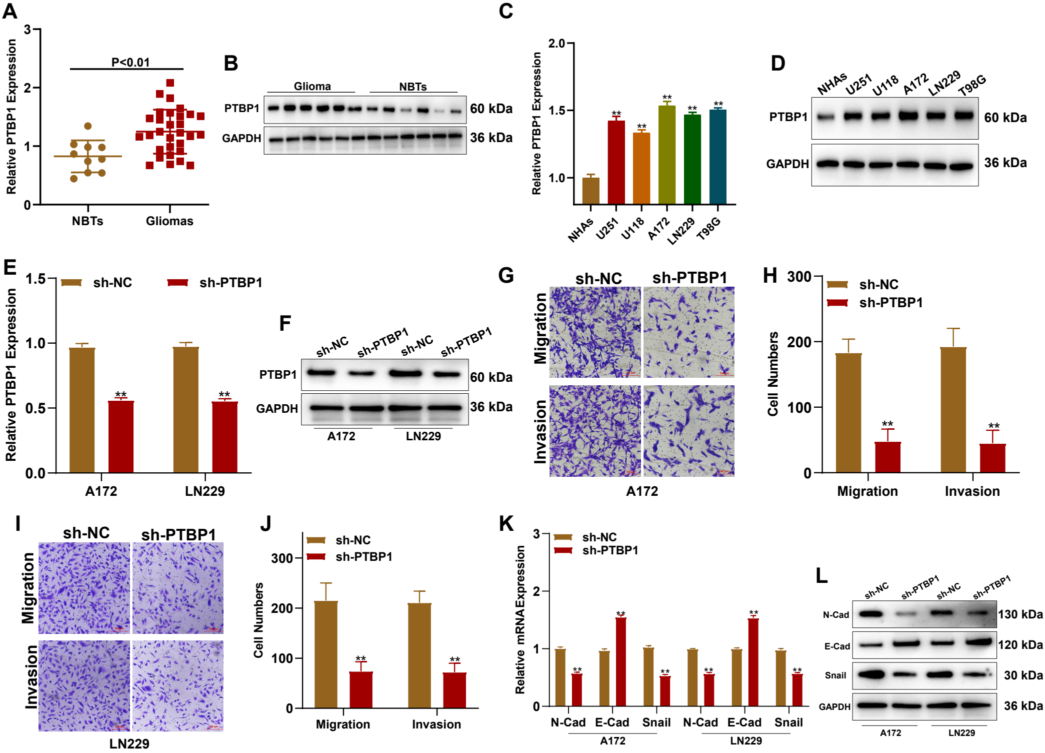

A
Relative PTBP1 Expression
P<0.01
NBTs
Gliomas
B
Glioma
NBTs
PTBP1
GAPDH
60 kDa
36 kDa
C
Relative PTBP1 Expression
NHAs
U251
U118
A172
LN229
T98G
D
PTBP1
GAPDH
60 kDa
36 kDa
E
sh-NC
sh-PTBP1
Relative PTBP1 Expression
A172
LN229
F
sh-NC
sh-PTBP1
PTBP1
GAPDH
60 kDa
36 kDa
A172
LN229
G
Migration
Invasion
A172
H
Cell Numbers
Migration
Invasion
I
LN229
J
Cell Numbers
K
Relative mRNA Expression
N-Cad
E-Cad
Snail
L
130 kDa
120 kDa
30 kDa

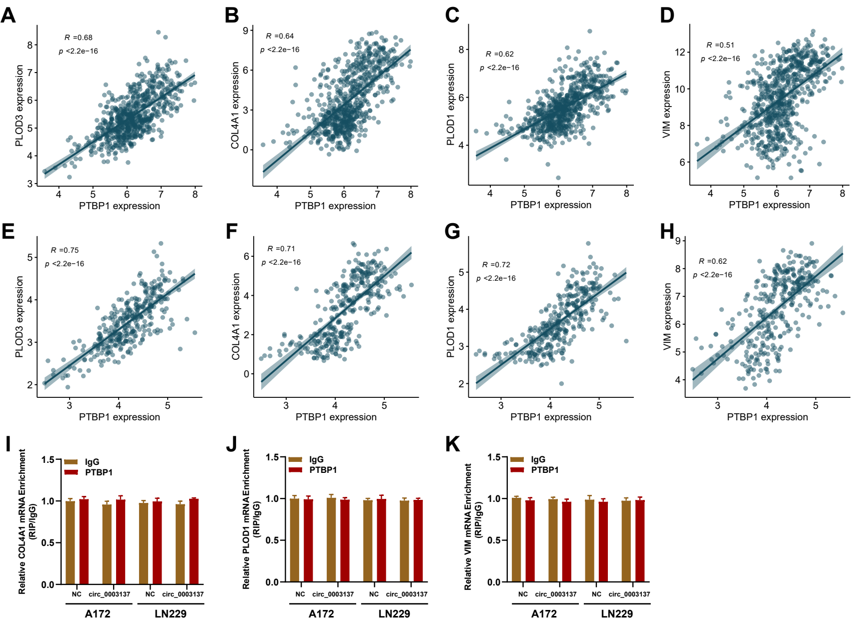

A
R =0.68
p <2.2e−16
PLOD3 expression
PTBP1 expression
B
R =0.64
p <2.2e−16
COL4A1 expression
PTBP1 expression
C
R =0.62
p <2.2e−16
PLOD1 expression
PTBP1 expression
D
R =0.51
p <2.2e−16
VIM expression
PTBP1 expression
E
R =0.75
p <2.2e−16
PLOD3 expression
PTBP1 expression
F
R =0.71
p <2.2e−16
COL4A1 expression
PTBP1 expression
G
R =0.72
p <2.2e−16
PLOD1 expression
PTBP1 expression
H
R =0.62
p <2.2e−16
VIM expression
PTBP1 expression
I
Relative COL4A1 mRNA Enrichment (RIP/IgG)
IgG
PTBP1
NC
circ_0003137
A172
LN229
J
Relative PLOD1 mRNA Enrichment (RIP/IgG)
IgG
PTBP1
NC
circ_0003137
A172
LN229
K
Relative VIM mRNA Enrichment (RIP/IgG)
IgG
PTBP1
NC
circ_0003137
A172
LN229

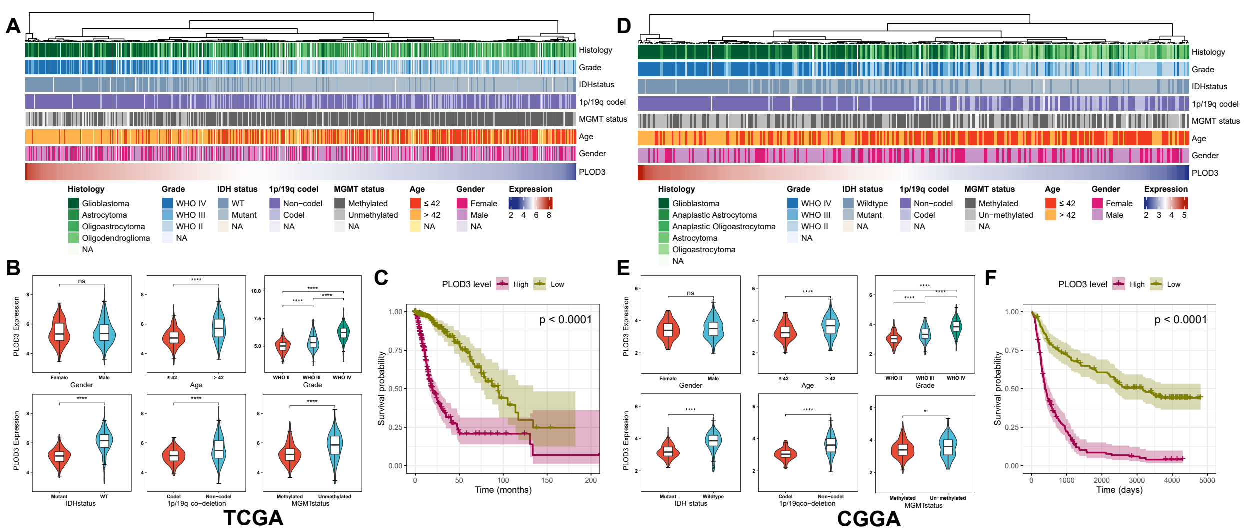

A
Histology
Grade
IDHstatus
1p/19q codel
MGMT status
Age
Gender
PLOD3
Histology
Glioblastoma
Astrocytoma
Oligoastrocytoma
Oligodendroglioma
NA
Grade
WHO IV
WHO III
WHO II
NA
IDH status
WT
Mutant
NA
1p/19q codel
Non−codel
Codel
NA
MGMT status
Methylated
Unmethylated
NA
Age
≤ 42
> 42
NA
Gender
Female
Male
NA
Expression
2 4 6 8
B
PLOD3 Expression
ns
Female
Male
Gender
****
≤ 42
> 42
Age
****
WHO II
WHO III
WHO IV
Grade
****
Mutant
WT
IDHstatus
****
Codel
Non−codel
1p/19q co−deletion
****
Methylated
Unmethylated
MGMTstatus
C
PLOD3 level
High
Low
p < 0.0001
Survival probability
Time (months)
TCGA
D
Histology
Grade
IDHstatus
1p/19q codel
MGMT status
Age
Gender
PLOD3
Histology
Glioblastoma
Anaplastic Astrocytoma
Anaplastic Oligoastrocytoma
Astrocytoma
Oligoastrocytoma
NA
Grade
WHO IV
WHO III
WHO II
NA
IDH status
Wildtype
Mutant
NA
1p/19q codel
Non−codel
Codel
NA
MGMT status
Methylated
Un−methylated
NA
Age
≤ 42
> 42
Gender
Female
Male
Expression
2 3 4 5
E
PLOD3 Expression
ns
Female
Male
Gender
****
≤ 42
> 42
Age
****
WHO II
WHO III
WHO IV
Grade
****
Mutant
Wildtype
IDH status
****
Codel
Non−codel
1p/19qco−deletion
*
Methylated
Un−methylated
MGMTstatus
F
PLOD3 level
High
Low
p < 0.0001
Survival probability
Time (days)
CGGA

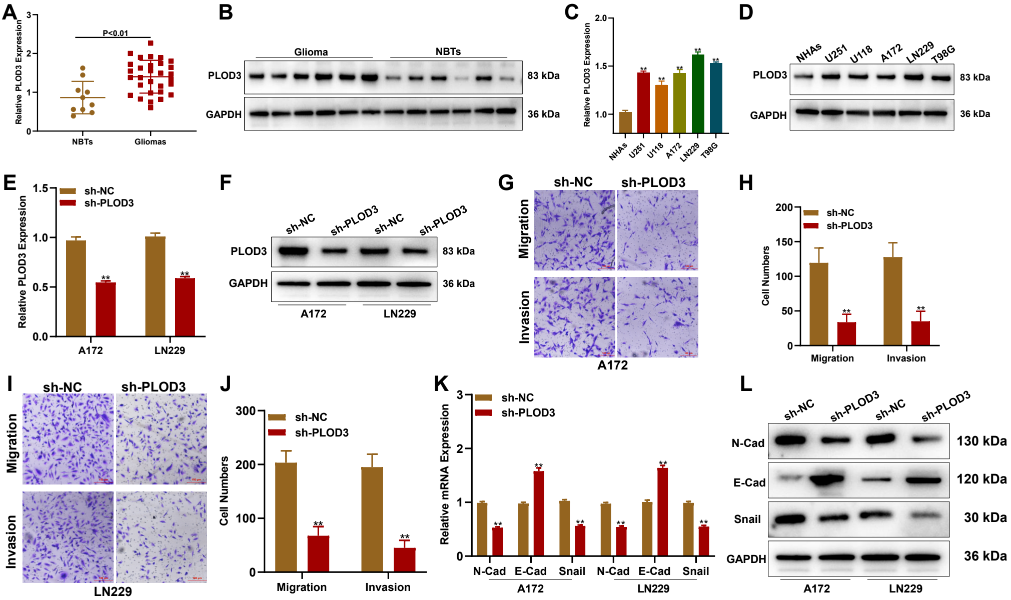
A
Relative PLOD3 Expression
P<0.01
NBTs
Gliomas
B
Glioma
NBTs
PLOD3
GAPDH
83 kDa
36 kDa
C
Relative PLOD3 Expression
NHAs
U251
U118
A172
LN229
T98G
D
NHAs
U251
U118
A172
LN229
T98G
PLOD3
GAPDH
83 kDa
36 kDa
E
sh-NC
sh-PLOD3
Relative PLOD3 Expression
A172
LN229
F
sh-NC
sh-PLOD3
sh-NC
sh-PLOD3
PLOD3
GAPDH
83 kDa
36 kDa
A172
LN229
G
sh-NC
sh-PLOD3
Migration
Invasion
A172
H
sh-NC
sh-PLOD3
Cell Numbers
Migration
Invasion
I
sh-NC
sh-PLOD3
Migration
Invasion
LN229
J
sh-NC
sh-PLOD3
Cell Numbers
Migration
Invasion
K
sh-NC
sh-PLOD3
Relative mRNA Expression
N-Cad
E-Cad
Snail
N-Cad
E-Cad
Snail
A172
LN229
L
sh-NC
sh-PLOD3
sh-NC
sh-PLOD3
N-Cad
E-Cad
Snail
GAPDH
130 kDa
120 kDa
30 kDa
36 kDa
A172
LN229

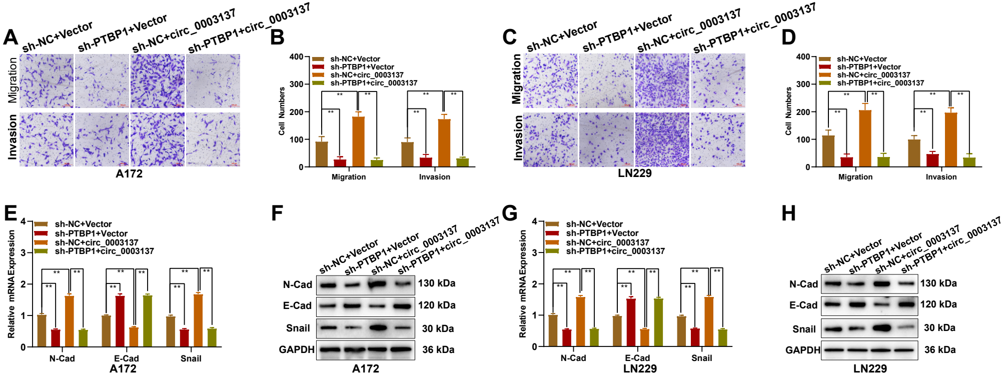
A
sh-NC+Vector
sh-PTBP1+Vector
sh-NC+circ_0003137
sh-PTBP1+circ_0003137
Migration
Invasion
A172
B
Cell Numbers
Migration
Invasion
C
LN229
D
E
Relative mRNA Expression
N-Cad
E-Cad
Snail
F
130 kDa
120 kDa
30 kDa
GAPDH
36 kDa
G
H

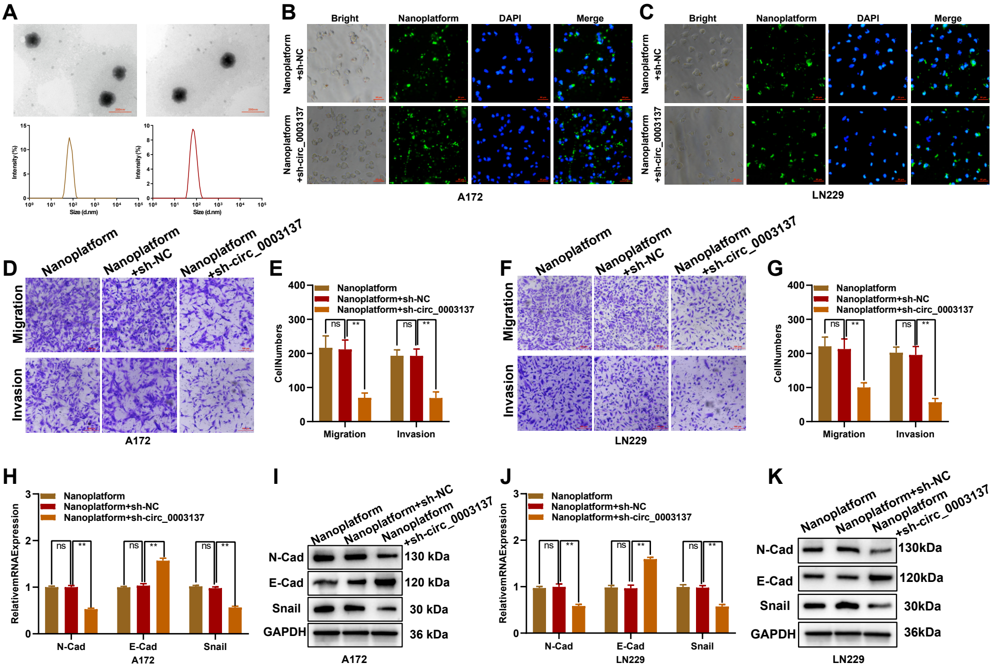
A
B
Bright
Nanoplatform
DAPI
Merge
Nanoplatform +sh-NC
Nanoplatform +sh-circ_0003137
A172
C
LN229
Intensity (%)
Size (d.nm)
D
Nanoplatform
Nanoplatform +sh-NC
Nanoplatform +sh-circ_0003137
Migration
Invasion
A172
E
Nanoplatform
Nanoplatform+sh-NC
Nanoplatform+sh-circ_0003137
CellNumbers
ns
**
Migration
Invasion
F
LN229
G
H
RelativemRNAExpression
N-Cad
E-Cad
Snail
A172
I
N-Cad
E-Cad
Snail
GAPDH
130 kDa
120 kDa
30 kDa
36 kDa
J
LN229
K
130kDa
120kDa
30kDa
36kDa

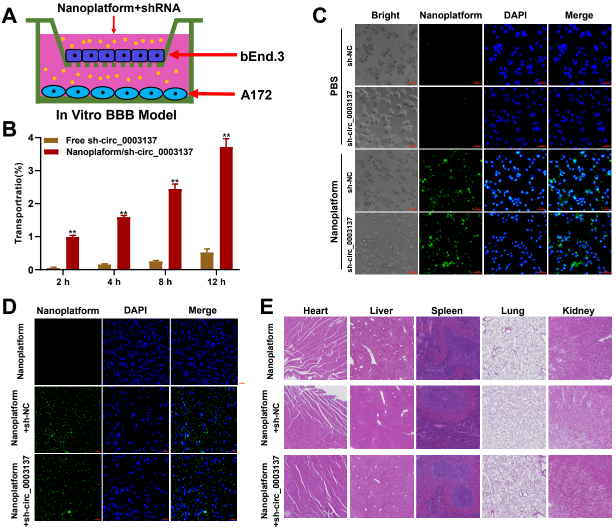
A
Nanoplatform+shRNA
bEnd.3
A172
In Vitro BBB Model
B
Free sh-circ_0003137
Nanoplaform/sh-circ_0003137
Transportratio(%)
2 h
4 h
8 h
12 h
C
Bright
Nanoplatform
DAPI
Merge
PBS
sh-NC
sh-circ_0003137
Nanoplatform
D
Nanoplatform
DAPI
Merge
Nanoplatform
+sh-NC
+sh-circ_0003137
E
Heart
Liver
Spleen
Lung
Kidney

The primers used in this study.

Gene name	Forward Sequence	Reverse Sequence
GAPDH	GTCTCCTCTGACTTCAACAGCG	ACCACCCTGTTGCTGTAGCCAA
circ_0003137	CTGAGGAGCAGCTTCAGTCC	GGCCATGTCCAACTCCATCA
PTBP1	CTCCAAGTTCGGCACAGTGTTG	CAGGCGTTGTAGATGTTCTGCC
PLOD3	CGAGTGTGAGTTCTACTTCAGCC	CCAGAAGTTGGACCACAGCTTG
CTNNB1	CACAAGCAGAGTGCTGAAGGTG	GATTCCTGAGAGTCCAAAGACAG
CD68	CGAGCATCATTCTTTCACCAGCT	ATGAGAGGCAGCAAGATGGACC
CD163	CCAGAAGGAACTTGTAGCCACAG	CAGGCACCAAGCGTTTTGAGCT
CD206	AGCCAACACCAGCTCCTCAAGA	CAAAACGCTCGCGCATTGTCCA
CD86	CCATCAGCTTGTCTGTTTCATTCC	GCTGTAATCCAAGGAATGTGGTC
TNF-α	CTCTTCTGCCTGCTGCACTTTG	ATGGGCTACAGGCTTGTCACTC
HIF1α	TATGAGCCAGAAGAACTTTTAGGC	CACCTCTTTTGGCAAGCATCCTG
COL4A1	TGTTGACGGCTTACCTGGAGAC	GGTAGACCAACTCCAGGCTCTC
PLOD1	GCCGTTTGTGTCCCTGTTCTTC	ATGCTGTGCCAGGAACTCTTCC
VIM	AGGCAAAGCAGGAGTCCACTGA	ATCTGGCGTTCCAGGGACTCAT
N-Cad	CCTCCAGAGTTTACTGCCATGAC	GTAGGATCTCCGCCACTGATTC
E-Cad	GCCTCCTGAAAAGAGAGTGGAAG	TGGCAGTGTCTCTCCAAATCCG
Snail	TGCCCTCAAGATGCACATCCGA	GGGACAGGAGAAGGGCTTCTC

The antibodies used in Western blot assay.

Protein name	Company	Catalogue number
CD68	Abcam	ab303565
CD163	Proteintech	16646-1-AP
CD206	Proteintech	18704-1-AP
CD86	Proteintech	13395-1-AP
TNF-α	Proteintech	60291-1-Ig
HIF1α	Proteintech	66730-1-Ig
GAPDH	Proteintech	60004-1-Ig
PTBP1	Proteintech	67462-1-Ig
PLOD3	Proteintech	11027-1-AP
CTNNB1	Proteintech	66379-1-Ig
N-Cad	Proteintech	66219-1-Ig
E-Cad	Proteintech	20874-1-AP
Snail	Proteintech	13099-1-AP
CD63	Proteintech	25682-1-AP
CD81	Proteintech	66866-1-Ig
Calnexin	Proteintech	10427-2-AP
TSG101	Proteintech	67381-1-Ig

Epithelial-mesenchymal transition hallmark genes set

ABI3BP	ACTA2	ADAM12	ANPEP	APLP1
AREG	BASP1	BDNF	BGN	BMP1
CADM1	CALD1	CALU	CAP2	CAPG
CD44	CD59	CDH11	CDH2	CDH6
COL11A1	COL12A1	COL16A1	COL1A1	COL1A2
COL3A1	COL4A1	COL4A2	COL5A1	COL5A2
COL5A3	COL6A2	COL6A3	COL7A1	COL8A2
COMP	COPA	CRLF1	CTGF	CTHRC1
CXCL1	CXCL12	CXCL6	CYR61	DAB2
DCN	DKK1	DPYSL3	DST	ECM1
ECM2	EDIL3	EFEMP2	ELN	EMP3
ENO2	FAP	FAS	FBLN1	FBLN2
FBLN5	FBN1	FBN2	FERMT2	FGF2
FLNA	FMOD	FN1	FOXC2	FSTL1
FSTL3	FUCA1	FZD8	GADD45A	GADD45B
GAS1	GEM	GJA1	GLIPR1	GLT25D1
GPC1	GPX7	GREM1	HTRA1	ID2
IGFBP2	IGFBP3	IGFBP4	IL15	IL32
IL6	IL8	INHBA	ITGA2	ITGA5
ITGAV	ITGB1	ITGB3	ITGB5	JUN
LAMA1	LAMA2	LAMA3	LAMC1	LAMC2
LEPRE1	LGALS1	LOX	LOXL1	LOXL2
LRP1	LRRC15	LUM	MAGEE1	MATN2
MATN3	MCM7	MEST	MFAP5	MGP
MMP1	MMP14	MMP2	MMP3	MSX1
MXRA5	MYL9	MYLK	NID2	NNMT
NOTCH2	NT5E	NTM	OXTR	PCOLCE
PCOLCE2	PDGFRB	PDLIM4	PFN2	PLAUR
PLOD1	PLOD2	PLOD3	PMEPA1	PMP22
POSTN	PPIB	PRRX1	PRSS2	PTHLH
PTX3	PVR	QSOX1	RGS4	RHOB
SAT1	SCG2	SDC1	SDC4	SERPINE1
SERPINE2	SERPINH1	SFRP1	SFRP4	SGCB
SGCD	SGCG	SLC6A8	SLIT2	SLIT3
SNAI2	SNTB1	SPARC	SPOCK1	SPP1
TAGLN	TFPI2	TGFB1	TGFBI	TGFBR3
TGM2	THBS1	THBS2	THY1	TIMP1
TIMP3	TNC	TNFAIP3	TNFRSF11B	TNFRSF12A
TPM1	TPM2	TPM4	VCAM1	VCAN
VEGFA	VEGFC	VIM	WIPF1	WNT5A

Note: The data derived from https://www.gsea-msigdb.org/gsea/msigdb/human/geneset/

The target genes of PTBP1 which screened according to their expression levels (based on TCGA)

Gene name	HR (hazard ratio)	Pearson Correlation
SERPINH1	6	0.49
TNFRSF12A	5.6	0.43
COL5A2	5.3	0.41
COL4A1	5.1	0.55
CALU	5.1	0.5
CAPG	5	0.31
PLOD3	4.8	0.59
FN1	4.7	0.27
PLOD1	4.6	0.53
VIM	4.6	0.53
MMP14	4.6	0.48
FLNA	4.5	0.53
MEST	4.3	0.47
LAMC1	4.1	0.55
LOXL2	4	0.25
COL3A1	4	0.16
SFRP4	3.9	0.27
PMP22	3.8	0.28
CD44	3.5	0.36
MGP	3.5	0.15
TNC	3.4	0.51
PPIB	3.3	0.51
ITGB1	3.3	0.48
MMP2	3.3	0.46
PLOD2	3.1	0.41
IGFBP3	3.1	0.29
TGFBI	3.1	0.19
ECM2	2.7	0.19
GAS1	2.5	0.54
GEM	2.4	0.23
TGFB1	2.3	0.36
ITGB5	2.1	0.27
CDH11	1.9	0.49
CDH2	1.8	0.65
PCOLCE2	1.8	0.28
MCM7	1.7	0.63
MATN2	1.7	0.32
SGCB	1.7	0.11
WNT5A	1.6	0.33
NT5E	1.5	0.39
ITGAV	1.5	0.31

WIPF1	1.5	0.3
FERMT2	1.3	0.46
SNTB1	1.3	0.31
ID2	1.2	0.23
DAB2	1.2	0.17
LRP1	1.1	0.38
NOTCH2	0.98	0.32
PRRX1	0.85	0.33
HTRA1	0.73	-0.11
RHOB	0.65	-0.11
VCAN	0.57	0.19
DPYSL3	0.48	0.11
TIMP3	0.4	0.042
ABI3BP	0.4	-0.064
